# Constitutive deletion of the obscurin-Ig58/59 domains induces atrial remodeling and Ca^2+^-based arrhythmogenesis

**DOI:** 10.1172/jci.insight.184202

**Published:** 2025-01-07

**Authors:** Alyssa Grogan, Annie Brong, Humberto C. Joca, Liron Boyman, Aaron D. Kaplan, Christopher W. Ward, Maura Greiser, Aikaterini Kontrogianni-Konstantopoulos

**Affiliations:** 1 Department of Biochemistry and Molecular Biology;; 2 Department of Orthopedics;; 3 Department of Physiology and Center for Biomedical Engineering and Technology;; 4 Marlene and Stewart Greenebaum Comprehensive Cancer Center;; 5 Division of Cardiology, Department of Medicine; and; 6 Claude D. Pepper Older Americans Independence Center, University of Maryland School of Medicine, Baltimore, Maryland, USA.

**Keywords:** Muscle biology, Arrhythmias, Calcium signaling, Cardiovascular disease

## Abstract

Obscurin is a giant protein that coordinates diverse aspects of striated muscle physiology. Obscurin immunoglobulin domains 58/59 (Ig58/59) associate with essential sarcomeric and Ca^2+^ cycling proteins. To explore the pathophysiological significance of Ig58/59, we generated the *Obscn-ΔIg58/59* mouse model, expressing obscurin constitutively lacking Ig58/59. Males in this line develop atrial fibrillation by 6 months, with atrial and ventricular dilation by 12 months. As *Obscn-ΔIg58/59* left ventricles at 6 months exhibit no deficits in sarcomeric ultrastructure or Ca^2+^ signaling, we hypothesized that susceptibility to arrhythmia may emanate from the atria. Ultrastructural evaluation of male *Obscn-ΔIg58/59* atria uncovered prominent Z-disk streaming by 6 months and further misalignment by 12 months. Relatedly, isolated *Obscn-ΔIg58/59* atrial cardiomyocytes exhibited increased Ca^2+^ spark frequency and age-specific alterations in Ca^2+^ cycling dynamics, coinciding with arrhythmia onset and progression. Quantitative analysis of the transverse-axial tubule (TAT) network using super-resolution microscopy demonstrated significant TAT depletion in *Obscn-ΔIg58/59* atria. These structural and Ca^2+^ signaling deficits were accompanied by age-specific alterations in the expression or phosphorylation of T-cap protein, which links transverse tubules to Z-disks, and junctophilin 2, which connects transverse tubules to the sarcoplasmic reticulum. Collectively, our work establishes the *Obscn-ΔIg58/59* model as a reputable genetic model for atrial cardiomyopathy and provides mechanistic insights into atrial fibrillation and remodeling.

## Abstract 2

Our genetic murine model (Obscn-ΔIg58/59) reliably recapituales the hallmarks of atrial cardiomyopathy and can be used as a reputable proxy to study disease pathogenicity, which remains largely unknown.

## Introduction

Atrial cardiomyopathy (ACM) is a complex disease that unifies etiologically distinct dysfunction initiated in the upper chambers of the heart. It was codified into histopathological classes in 2016 by the European Heart Rhythm Association, characterized by contractile, interstitial, and electrophysiological alterations, and divided into 4 nonhierarchical classes (1). While ACM is a highly heterogeneous condition with respect to phenotypes and causative forces, atrial fibrillation (AF) and dilation are hallmarks of the disease shared across all 4 classes of ACM (1). AF is the most common arrhythmia worldwide and is associated with high morbidity and mortality (2). Changes in atrial conduction, shortening of the atrial action potential, and ectopic focal activity contribute to AF (3, 4). Importantly, a positive feedback loop exists where the structural and electrical remodeling that begets AF is perpetuated by prolonged AF, intrinsically escalating ACM (1, 3).

Given the recent recognition of ACM as a widespread clinical entity, basic science is unequipped with analogous model systems. Several animal models of AF involve exogenous intervention, such as electrode implantation for rapid atrial pacing, vagal interference, or surgery that abruptly initiate episodes of fibrillation (5, 6). These models often fail to mimic the progressive nature of AF, which gradually advances from a paroxysmal to persistent presentation. Furthermore, some surgical models and many genetic mouse models that feature AF have a preeminent ventricular cardiomyopathy or heart failure, such that atrial disturbances may be ancillary (3). Accordingly, there are few, primarily large, animal models where structural and electrical remodeling of the atria is clearly antecedent to or independent of ventricular dysfunction (7). Here, we present a mouse model that phenocopies key features of human AF, generated by the deletion of 2 domains within the *OBSCN* gene.

Obscurin (720–870 kDa), encoded by the *OBSCN* gene, is a giant protein harboring both cytoskeletal and signaling modalities that encircles myofibrils along M-bands and Z-disks. Obscurin serves essential roles in myofibrillar assembly, cell adhesion, Ca^2+^ signaling, and the integration of the sarcomere with the surrounding membrane and cytoskeletal structures (8, 9). Rare and deleterious variants in *OBSCN* have been increasingly associated with the development of cardiomyopathy in humans, as more than 20 missense, splicing, and frameshift mutations have been identified in patients with hypertrophic cardiomyopathy (HCM) and dilated cardiomyopathy, left ventricular noncompaction, and arrhythmogenic right ventricular cardiomyopathy (10–12). Although the pathophysiological consequences of most known *OBSCN* mutations remain unresolved, our lab previously generated the *Obscn-R4344Q* mouse model containing the HCM-linked point mutation, R4344Q, residing within immunoglobulin (Ig) domain 58 (13). Sedentary *Obscn-R4344Q* mice developed spontaneous ventricular arrhythmia by 12 months associated with increased Ca^2+^ cycling kinetics, linked to enhanced binding of phospholamban (PLN) to mutant Ig58-R4344Q (13).

The obscurin-Ig58/59 module interacts with indispensable regulators of muscle structure and function, including PLN, the Z-disk–localized NH_2_ terminal region of titin (3–4 MDa), and the titin splice variant novex-3 (~700 kDa) (13–15). Consequently, we generated the *Obscn-ΔIg58/59* mouse model that expresses endogenous obscurin constitutively lacking Ig58/59 (16). Our initial characterization revealed that sedentary *Obscn-ΔIg58/59* male animals develop spontaneous AF by 6 months that is greatly exacerbated by 12 months, when atrial enlargement and ventricular dilation also manifest (16). While a compensatory upregulation of the sarco-endoplasmic reticulum Ca^2+^ ATPase 2 (SERCA2) and its regulator PLN accompanied enhanced ventricular ejection fraction and fractional shortening in *Obscn-ΔIg58/59* animals at 6 months (16), no differences in ventricular myocyte contractility, Ca^2+^ transients, ultrastructure, and fibrotic infiltration were detected (16).

In pursuit of the mechanistic source of atrial arrhythmogenesis and remodeling in *Obscn-ΔIg58/59* mice, we investigated the structural and functional impact of the Ig58/59 deletion in atria at the cellular level. Herein, we show that *Obscn-ΔIg58/59* atria exhibit prominent ultrastructural deficits at the level of the Z-disk and the transverse-axial tubule (TAT) network. Intriguingly, Ca^2+^ cycling alterations occur in *Obscn-ΔIg58/59* atrial cardiomyocytes at 6 months, earlier than in ventricular cardiomyocytes, and progress by 12 months, coinciding with the onset and aggravation of arrhythmia (16). Mechanistically, *Obscn-ΔIg58/59* atrial dysfunction is associated with changes in the expression and phosphorylation profile of T-cap, a sarcomeric titin-binding protein that links transverse tubules to Z-disks, and junctophilin 2, which spans the cardiac dyad tethering transverse tubules to the sarcoplasmic reticulum. Collectively, our studies provide insights into the development of atrial remodeling and spontaneous AF that precede ventricular maladaptation.

## Results

### Ultrastructural evaluation of Obscn-ΔIg58/59 atria reveals prominent Z-disk streaming and misalignment.

**Figure 1:**
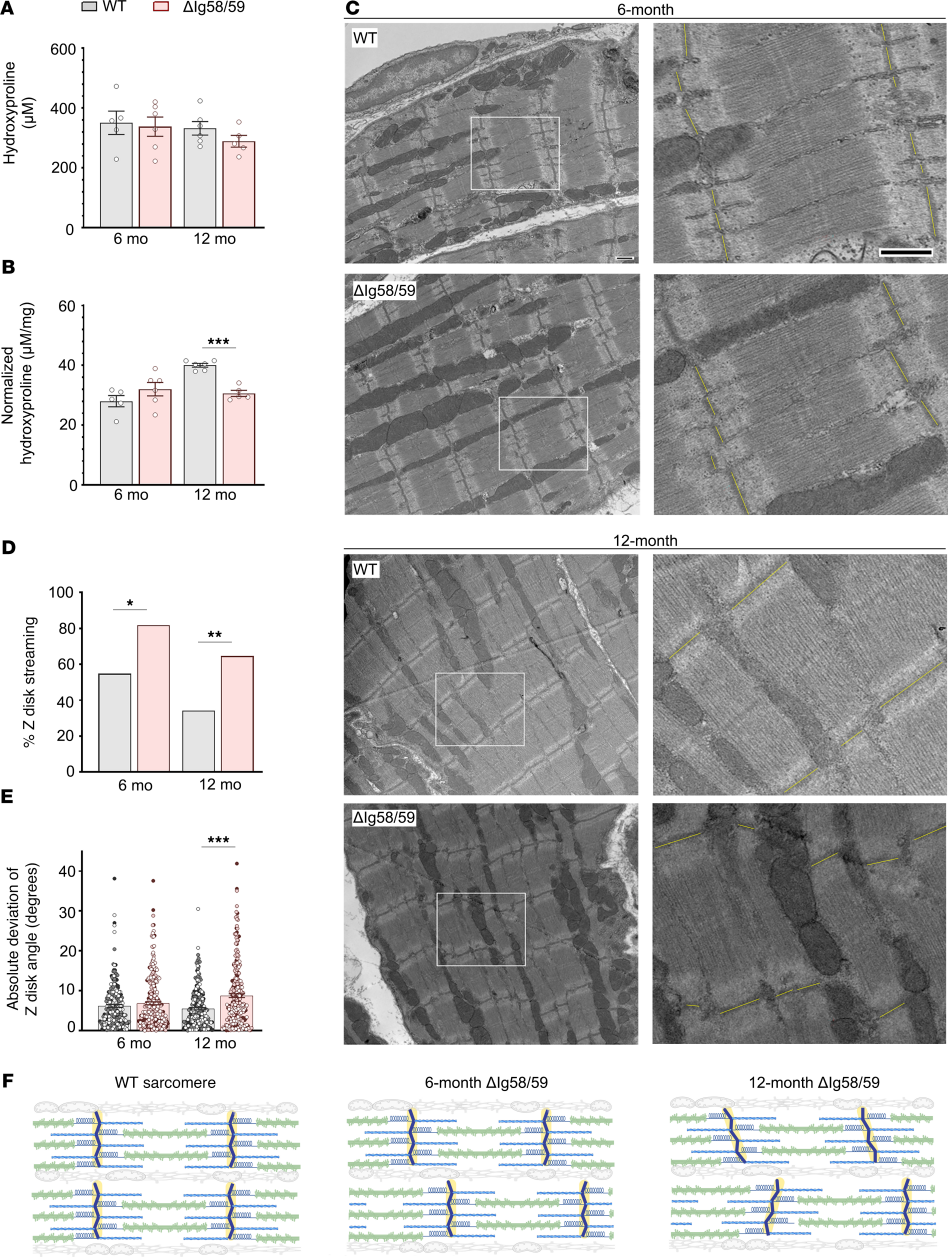
Ultrastructural analysis reveals Z-disk abnormalities in *Obscn-ΔIg58/59* atria. (**A** and **B**) Quantification of absolute hydroxyproline content (**A**) in atrial tissues did not reveal differences in fibrotic deposition between genotypes at 6 or 12 months. In contrast, when normalized to atrial tissue mass (**B**), hydroxyproline content was significantly reduced in *Obscn-ΔIg58/59* atria at 12 months compared with age-matched wild-type, indicating that the atrial enlargement observed in *Obscn-ΔIg58/59* mice at this time point is not associated with increased fibrosis; *t* test, ***P < 0.001; *n* = 5–6 animals per group; data points represent the average of 6 technical replicates per animal. (**C**) Representative electron micrographs of longitudinally sectioned atria depicted Z-disk streaming in *Obscn-ΔIg58/59* hearts at 6 and 12 months, along with increased variability in Z-disk orientation at 12 months; scale bar: 500 nm. Z-disks are highlighted in yellow in the images on the right, which are zoomed-in areas of the electron micrographs on the left, denoted by white rectangles. (**D**) The percentage of images that contained Z-disk streaming was significantly increased in 6- and 12-month *Obscn-ΔIg58/59* atria compared with controls; Fisher’s exact test, *P < 0.05, **P < 0.01; *n* = 3 animals per group, 10 ± 3 images per animal (6 months), 15 ± 3 images per animal (12 months). (**E**) *Obscn-ΔIg58/59* atrial sarcomeres displayed significantly increased variability in the orientation of the Z-disk at 12 months as quantified by the absolute deviation of the Z-disk angle within each image; *t* test, ***P < 0.001; *n* = 3 animals per group, 2 images per animal; data points represent individual sarcomeres and are color coded by biological replicate. (**F**) Schematic illustrating the progressive changes in Z-disk architecture in *Obscn-ΔIg58/59*. While wild-type sarcomeres are properly aligned, Z-disks in *Obscn-ΔIg58/59* atria are out of register (i.e., Z-disk streaming) by 6 months and nonparallel by 12 months. Figure generated with BioRender.com (License MB27PC5ZUC).

Our initial characterization of the *Obscn-*Δ*Ig58/59* model revealed spontaneous AF in 6-month-old *Obscn-*Δ*Ig58/59* male animals that progressed in severity by 12 months, accompanied by gross atrial enlargement (16). While fibrosis is a known driver of alterations in electrical conduction and force production in atria (1), *Obscn-*Δ*Ig58/59* atria did not exhibit increased absolute fibrotic content at 6 or 12 months compared to age-matched wild-type (Figure 1A). Interestingly, when normalized to total atrial mass, fibrotic content was significantly decreased in 12-month-old *Obscn-*Δ*Ig58/59* atria compared with age-matched wild-type (Figure 1B). This indicates that the gross atrial enlargement manifesting at this time point (16) is not accompanied by fibrotic deposition, eliminating fibrosis as a possible mechanistic source of arrhythmogenesis.

Our previous biochemical analysis did not indicate differences in the expression levels of obscurin, the binding partners of the Ig58/59 module (titin and PLN), or canonical Ca^2+^ handling proteins between wild-type and *Obscn-*Δ*Ig58/59* atria at 6 or 12 months of age (17). Proteomics/phosphoproteomics analysis, however, exposed extensive changes in the expression and phosphorylation profile of Z-disk–associated cytoskeletal proteins and Ca^2+^ cycling regulators in *Obscn-*Δ*Ig58/59* atria at both 6 and 12 months of age, highlighting proteins and phosphorylation events with uncharacterized (patho)physiological roles in the heart (17) that could drive *Obscn-*Δ*Ig58/59* atrial remodeling and dysfunction.

Considering the plethora of deregulated cytoskeletal proteins in *Obscn-*Δ*Ig58/59* atria (17), we evaluated sarcomeric ultrastructure using electron microscopy. Although there were no obvious abnormalities in overall sarcomeric organization, we observed significant Z-disk streaming at both 6 and 12 months (Figure 1, C and D), a common myopathic manifestation characterized by out-of-register Z-disks (18), indicative of lateral myofibrillar misalignment or structural deficiency of the Z-disk itself. Moreover, *Obscn-*Δ*Ig58/59* atria displayed increased variability in Z-disk orientation at 12 months, as determined by the absolute deviation of the Z-disk angle of individual sarcomeres (Figure 1E). Given that Z-disk alignment remained unaffected in *Obscn-*Δ*Ig58/59* left ventricles (16), these findings indicated that *Obscn-*Δ*Ig58/59* atria are more susceptible to developing structural defects, particularly impacting Z-disk placement and orientation (Figure 1F). Since the Z-disk is a structural and signaling hub bridging the sarcomere to the extra-sarcomeric cytoskeleton and the neighboring internal membrane systems (i.e., the TAT network and the sarcoplasmic reticulum, SR), these results are consistent with our proteomics study that revealed deregulation of proteins in each of these subcellular compartments (17). It is therefore plausible that the obscurin-Ig58/59 module stabilizes Z-disk–associated protein complexes and supports the overall alignment of adjacent sarcomeres and surrounding structures in atria.

### Atrial cardiomyocytes from sedentary Obscn-ΔIg58/59 males exhibit elevated Ca^2+^ spark frequency and age-specific changes in intracellular Ca^2+^ cycling.

**Figure 2:**
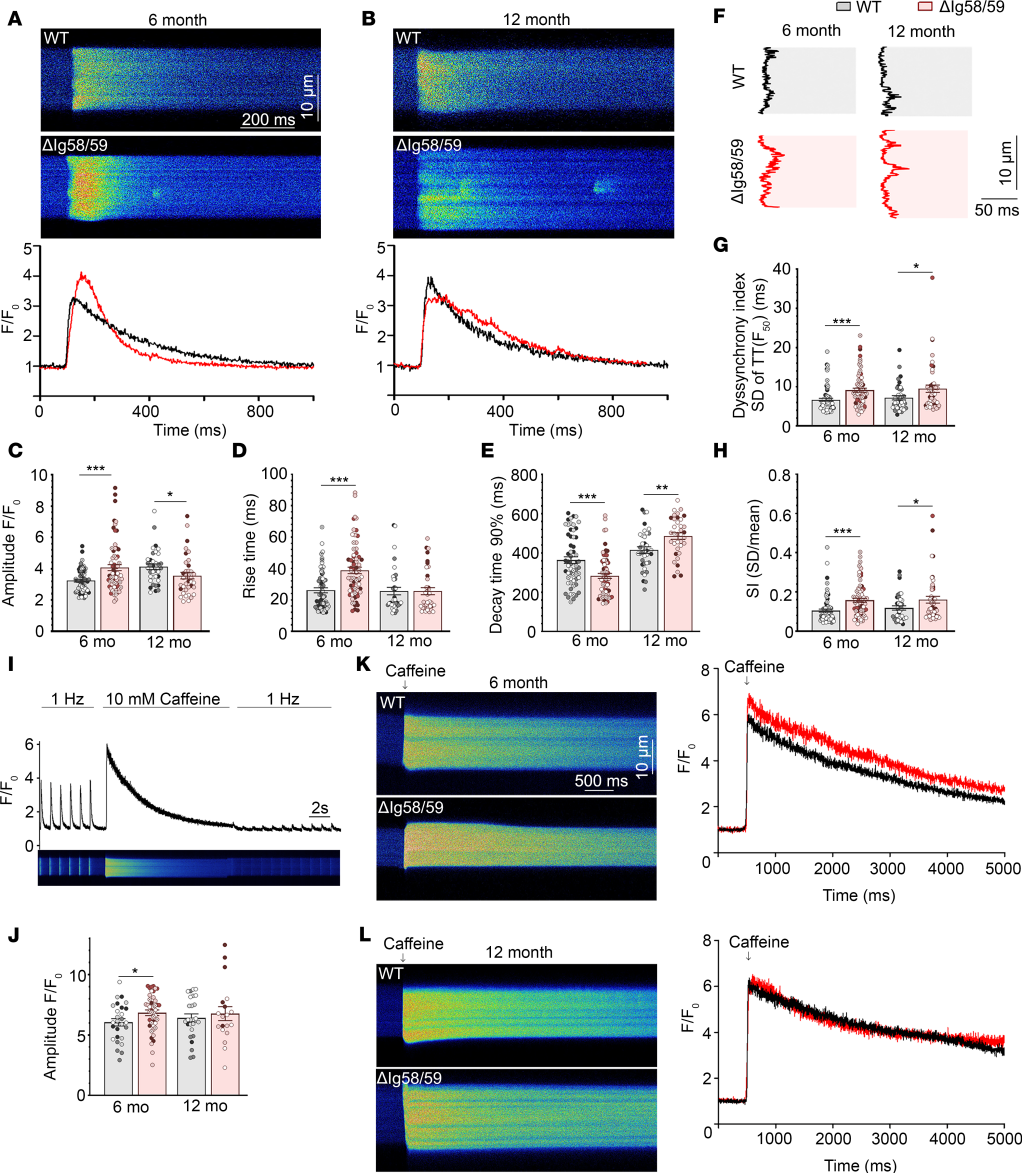
Atrial cardiomyocytes from sedentary *Obscn-ΔIg58/59* mice exhibit age-specific changes in Ca^2+^ cycling and SR Ca^2+^ content. (**A**–**E**) Representative confocal line scan images and corresponding Ca^2+^ transients (**A** and **B**) depicted significantly increased Ca^2+^ transient amplitude (**C**) and rise time (**D**) and decreased Ca^2+^ decay time (**E**) in atrial cardiomyocytes from 6-month-old *Obscn-ΔIg58/59* hearts compared with age-matched wild-type, whereas 12-month-old *Obscn-ΔIg58/59* cells displayed significantly decreased Ca^2+^ transient amplitude (**C**) and prolonged Ca^2+^ decay (**E**) compared with controls, with no change in rise time (**D**); *t* test, *P < 0.05, **P < 0.01, ***P < 0.001; *n* = 5 animals per group (6 months), *n* = 3 animals per group (12 months), 9–20 cells per heart (6 months), 7–17 cells per heart (12 months); data points represent individual cells color coded by biological replicate. (**F**) Line profiles of the representative Ca^2+^ transients depicted in **A** and **B** at half-maximal fluorescence amplitude and corresponding quantifications of the SD of the TTF_50_ (**G**) and the coefficient of variation, SI (**H**), revealed dyssynchronous Ca^2+^ release in *Obscn-*Δ*Ig58/59* atria at 6 and 12 months; *t* test, *P < 0.05, ***P < 0.001; *n* = 5 animals per group (6 months), *n* = 3–4 animals per group (12 months), 6–24 cells per heart (6 months), 2–17 cells per heart (12 months); data points represent individual cells color coded by biological replicate. (**I**–**L**) SR Ca^2+^ load was measured in quiescent atrial cardiomyocytes via rapid application of 10 mmol/L caffeine preceded by steady-state 1 Hz electrical pacing. Field stimulation was subsequently restarted to ensure that all releasable Ca^2+^ had been depleted (**I**). Representative transverse confocal line scan images and caffeine-induced Ca^2+^ transients at 6 months (**K**) and 12 months (**L**) depicted significantly increased SR Ca^2+^ content (**J**) in atrial cardiomyocytes isolated from 6-month-old *Obscn-ΔIg58/59* hearts, but not at 12 months, compared with age-matched controls; *t* test, *P < 0.05; *n* = 4–5 animals per group (6 months), *n* = 3–4 animals per group (12 months), 5–11 cells per heart (6 months), 2–11 cells per heart (12 months); data points represent individual cells color coded by biological replicate.

The presence of severe AF in *Obscn-*Δ*Ig58/59* mice (16) and the prominent alterations in key regulators of intracellular Ca^2+^ cycling identified in our phospho-proteomic screen (17) prompted us to further evaluate Ca^2+^ homeostasis in atrial cardiomyocytes isolated from sedentary *Obscn-*Δ*Ig58/59* male animals at 6 and 12 months. Cardiomyocytes obtained from *Obscn-*Δ*Ig58/59* atria were moderately enlarged compared with wild-type at 6 months (*P* = 0.08), which progressed to significance by 12 months of age (Supplemental Figure 1, A and B; supplemental material available online with this article; https://doi.org/10.1172/jci.insight.184202DS1). To assess intracellular Ca^2+^ cycling dynamics, Ca^2+^ transients were measured in freshly isolated atrial cardiomyocytes. To ensure steady-state Ca^2+^ cycling conditions, atrial cardiomyocytes were electrically paced using field stimulation at a rate of 1 Hz. At 6 months, *Obscn-*Δ*Ig58/59* atrial cells displayed significantly increased Ca^2+^ transient amplitude and rise time, whereas Ca^2+^ decay time was significantly reduced compared with age-matched wild-type cells (Figure 2, A–E). Conversely, 12-month-old *Obscn-*Δ*Ig58/59* atrial cells exhibited significantly decreased Ca^2+^ transient amplitude and prolonged Ca^2+^ decay, while Ca^2+^ transient rise time was unaffected compared to controls (Figure 2, A–E). Importantly, quantification of the SD and the coefficient of variation (SI) of the time to half-maximal fluorescence (TTF_50_) assessing the spatial coordination of Ca^2+^ release along the width of cardiomyocytes demonstrated markedly desynchronized Ca^2+^ transients in *Obscn-*Δ*Ig58/59* atria at both 6 and 12 months (Figure 2, F–H).

SR Ca^2+^ content was also assessed by determining the amount of releasable Ca^2+^ following the application of caffeine after cells had been electrically stimulated at 1 Hz for 30 seconds to achieve steady-state SR Ca^2+^ loading. The amount of releasable SR Ca^2+^ was significantly elevated in *Obscn-*Δ*Ig58/59* atrial cardiomyocytes at 6 months, as evidenced by increased amplitude of caffeine-induced Ca^2+^ transients, whereas SR Ca^2+^ content was unaffected at 12 months (Figure 2, I–L). Taken together, these changes in intracellular Ca^2+^ cycling align with the natural progression of AF, where 6-month *Obscn-*Δ*Ig58/59* atrial cardiomyocytes show elevated SR load, increased and prolonged Ca^2+^ release, and faster Ca^2+^ decay kinetics, while SR load and kinetics at 12 months are depressed or unchanged. These alterations, along with the dyssynchronous Ca^2+^ release observed at both time points, are consistent with the progressive Ca^2+^ cycling defects typically associated with AF-induced remodeling and maladaptive Ca^2+^ signaling in ACM (1, 19).

**Figure 3:**
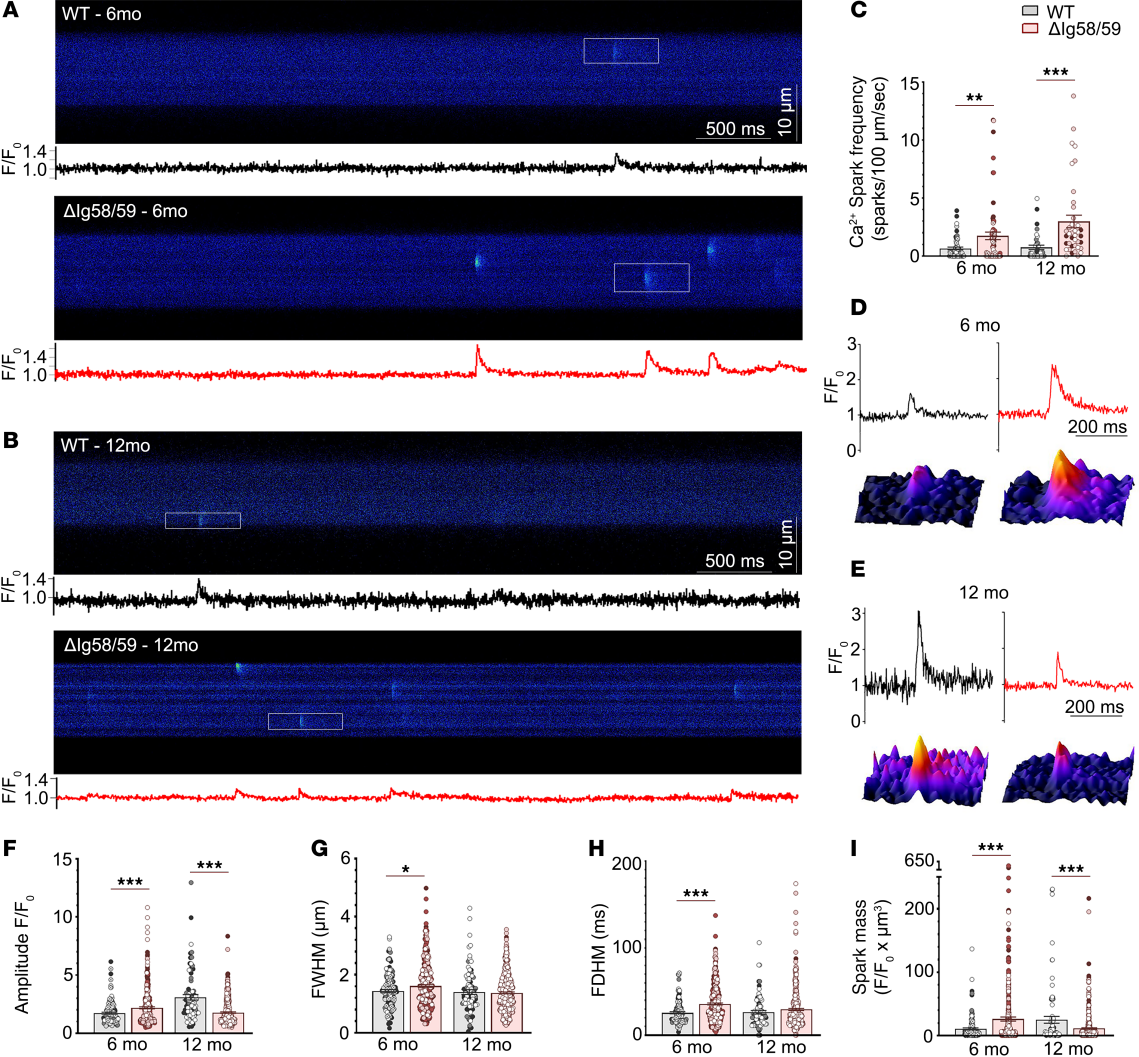
Elevated Ca^2+^ spark frequency in atrial cardiomyocytes from *Obscn-ΔIg58/59* mice. (**A** and **B**) Representative confocal line scan traces and corresponding fluorescence intensity profiles of unstimulated wild-type and *Obscn-ΔIg5859* atrial cells at 6 months (**A**) and 12 months (**B**). (**C**) Cells isolated from *Obscn-ΔIg5859* atria displayed a ~2.6- and ~4.0-fold increase in spark frequency compared with wild-type at 6 and 12 months, respectively; *t* test, **P < 0.01, ***P < 0.001; data points represent individual cells and are color coded by biological replicate. (**D** and **E**) Fluorescence intensity profiles and corresponding 3-dimensional surface plots of representative Ca^2+^ sparks indicated by white rectangles in **A** and **B** from wild-type and *Obscn-ΔIg5859* atria at 6 months (**D**) and 12 months (**E**). (**F**–**I**) Ca^2+^ spark analysis revealed significantly increased spark amplitude (**F**), FWHM (**G**), FDHM (**H**), and spark mass (**I**) in 6-month-old *Obscn-ΔIg5859* cells compared with wild-type, whereas 12 -month *Obscn-ΔIg5859* cells displayed significantly decreased spark amplitude (**F**) and spark mass (**I**) with no changes in FWHM (**G**) or FDHM (**H**); *t* test, *P < 0.05, ***P < 0.001; *n* = 5 animals per group (6 months), *n* = 3 animals per group (12 months), 9–20 cells per heart (6 months), 7–17 cells per heart (12 months); data points represent individual sparks and are color coded by biological replicate.

Given the abnormalities in atrial Ca^2+^ cycling identified in vitro and the episodes of spontaneous AF in *Obscn-*Δ*Ig58/59* animals (16), we next evaluated the frequency and morphology of spontaneous Ca^2+^ sparks in atrial cardiomyocytes. Ca^2+^ sparks are elemental Ca^2+^ release events originating from a single cluster of ryanodine receptors (RyR2) (20, 21). Critically, increased diastolic Ca^2+^ leak resulting from high spontaneous Ca^2+^ spark frequency has been associated with the development of AF and ventricular arrhythmias (20, 22, 23). Indeed, 6-month-old *Obscn-*Δ*Ig58/59* atrial cells exhibited a ~2.6-fold increase in Ca^2+^ spark frequency compared with age-matched controls (Figure 3, A and C). Further assessment of Ca^2+^ spark morphology revealed significantly increased spark amplitude, full width at half-maximum (FWHM), full duration at half-maximum (FDHM), spark mass, time to peak, and Tau (exponential time constant of spark decay) with no alterations in the maximum steepness of spark upstroke in 6-month-old *Obscn-*Δ*Ig58/59* atrial cells compared with wild-type (Figure 3, D and F–I, and Supplemental Figure 1, C–G). Strikingly, by 12 months, *Obscn-*Δ*Ig58/59* atrial cardiomyocytes exhibited an approximately 4.0-fold increase in Ca^2+^ spark frequency compared with age-matched controls (Figure 3, B and C). Morphologically, Ca^2+^ sparks originating from 12-month-old *Obscn-*Δ*Ig58/59* atrial cells displayed decreased amplitude and spark mass, with no significant alterations in FWHM, FDHM, time to peak, maximum steepness of spark upstroke, or Tau (Figure 3, E–I, and Supplemental Figure 1, C–G).

Collectively, these findings indicate increased intracellular Ca^2+^ load in 6-month *Obscn-*Δ*Ig58/59* atrial cardiomyocytes, where elevated SR Ca^2+^ levels are associated with augmented Ca^2+^ transients and more frequent and larger Ca^2+^ sparks. Conversely, by 12 months, *Obscn-*Δ*Ig58/59* atrial cells exhibit depressed Ca^2+^ transients and kinetics in the absence of elevated SR Ca^2+^ load along with the presence of more frequent but lower magnitude Ca^2+^ sparks. Importantly, this increased spontaneous Ca^2+^ spark activity at both time points implies a persistent Ca^2+^ leak from the SR that could promote AF in *Obscn-*Δ*Ig58/59* mice.

### The TAT network is disrupted in Obscn-ΔIg58/59 atria.

**Figure 4:**
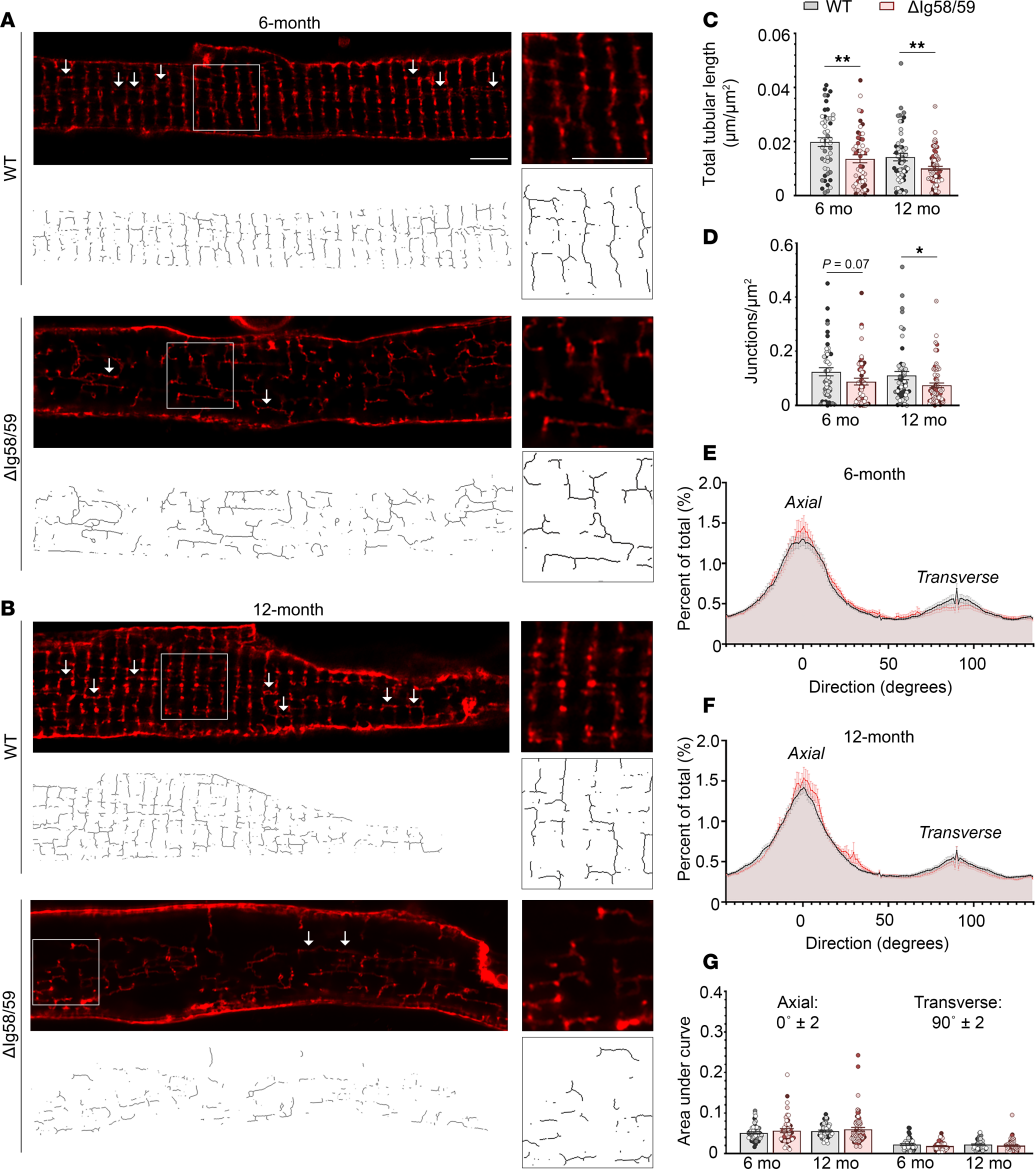
The transverse-axial tubule network is disrupted in *Obscn-ΔIg58/59* atria. (**A** and **B**) Representative super-resolution images of wild-type and *Obscn-ΔIg5859* atrial cardiomyocytes stained with di-8-ANEPPS at 6 months (**A**) and 12 months (**B**); arrows highlight axial tubule structures; scale bar: 5 μm. (**C** and **D**) The total length of the tubular network (**C**) is decreased in *Obscn-ΔIg58/59* atria at both 6 and 12 months, while the number of tubular junctions (**D**) is significantly diminished by 12 months; *t* test, *P < 0.05, **P < 0.01. (**E**–**G**) Histograms depicting the proportion of tubules at each orientation at 6 (**E**) and 12 (**F**) months and corresponding quantifications (**G**) did not indicate any differences in the distribution of axial and transverse tubules in *Obscn-ΔIg5859* cells compared with wild-type; *t* test, 0°: *P* = 0.34 (6 months), *P* = 0.43 (12 months), 90°: *P* = 0.13 (6 months), *P* = 0.27 (12 months); area under the curve was calculated within a range of ±2° from 0° (axial) or 90° (transverse). *n* = 4 animals per group (6 months), *n* = 4–6 animals per group (12 months), 9–14 cells per heart (6 months), 6–19 cells per heart (12 months); data points represent individual cells and are color coded by biological replicate.

The presence of progressive structural abnormalities at the level of the Z-disk along with age-specific alterations in Ca^2+^ cycling dynamics and Ca^2+^ spark frequency in *Obscn-*Δ*Ig58/59* atrial cells prompted us to investigate TAT membrane architecture using super-resolution microscopy. Freshly isolated, live atrial cardiomyocytes were stained with di-8-ANEPPS, a fluorescent lipophilic plasma membrane marker that is commonly used to visualize the TAT system (24, 25). Quantification of the length and orientation of the TAT network demonstrated a significant reduction in TAT density in *Obscn-*Δ*Ig58/59* atria at both 6 and 12 months compared with age-matched wild-type (Figure 4, A–D), with no significant changes in directionality (Figure 4, E–G). The observed structural deterioration of the TAT network in *Obscn-*Δ*Ig58/59* atria likely contributes to impaired Ca^2+^-induced Ca^2+^ release (CICR), resulting in asynchronous Ca^2+^ release from the SR and consequent arrhythmogenicity at both 6 and 12 months.

### The expression and phosphorylation status of T-cap is altered in Obscn-ΔIg58/59 atria.

To mechanistically interrogate the profound structural and Ca^2+^ cycling changes that we discovered in *Obscn-*Δ*Ig58/59* atria, we utilized our prior phosphoproteomics screen as a guide (17). Given the significant alterations in both Z-disk and TAT morphology in *Obscn-*Δ*Ig58/59* atria, we focused our investigation on T-cap, for which our phosphoproteomics analysis indicated altered phosphorylation in 12-month-old *Obscn-*Δ*Ig58/59* atria (17). T-cap binds to titin’s extreme NH_2_ terminal Ig1/2 domains located at the Z-disk in proximity to titin-Ig9/10 encompassing the binding site for obscurin-Ig58/59. T-cap at the Z-disk is postulated to support the structural integrity and physical association of the sarcomere with the transverse tubule network by interacting with ion channel accessory subunits (26–29), in addition to regulating responses to biomechanical and hemodynamic stress (30, 31). Endogenous T-cap exists in a constitutively biphosphorylated state at residues Ser157 and Ser161 (28). Although neither the hierarchy nor the function of each phosphorylation event is known, it has been postulated that the phosphorylation status of T-cap may regulate its susceptibility to proteasomal degradation and influence the integrity of the transverse tubule network in ventricular myocardium (28, 31, 32).

**Figure 5:**
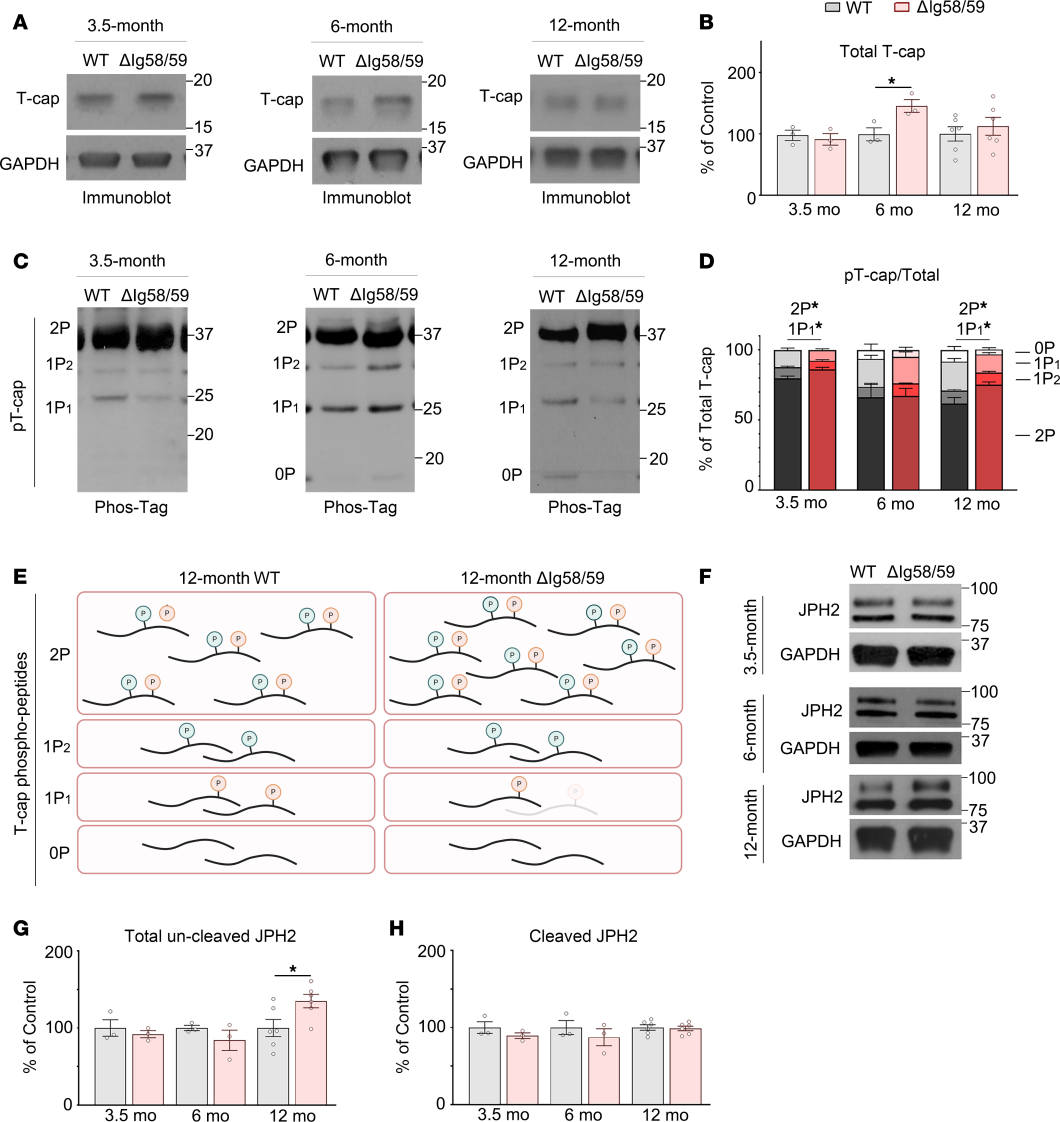
The expression and phosphorylation of T-cap are altered in *Obscn-ΔIg58/59* atria. (**A** and **B**) Representative immunoblots (**A**) and relative quantifications (**B**) revealed significantly increased T-cap expression in *Obscn-ΔIg5859* atria compared with wild-type at 6 months but not at 3.5 or 12 months. (**C** and **D**) Representative phosphorylated Phos-tag acrylamide immunoblots (**C**) and relative quantifications (**D**) did not indicate significant differences in normalized pT-cap at 6 months, but revealed increased levels of biphosphorylated T-cap (2P) and a corresponding decrease in the lower molecular weight monophosphorylated (1P_1_) T-cap species with no statistically significant differences in the higher molecular weight (1P_2_) or nonphosphorylated (0P) T-cap species in *Obscn-ΔIg5859* atria compared with wild-type at both 3.5 and 12 months. Nonphosphorylated T-cap species were not reliably detected at 3.5 months and therefore were not quantified; *t* test, *P < 0.05; (**A**): *n* = 3 animals per genotype for the 3.5- and 6-month time points and *n* = 6 animals per genotype for the 12-month time point; (**B**): *n* = 6 animals per genotype for the 3.5-month time point, *n* = 3 animals per genotype for the 6-month time point, and *n* = 5 animals per genotype for the 12-month time point; data points represent the average of at least 3 technical replicates per animal; quantifications of pT-cap were normalized to the summed intensity of all species for a given sample. (**E**) Schematic depicting the decrease in 1P_1_ T-cap species and corresponding increase in 2P T-cap observed in *Obscn-ΔIg5859* atria at 12 months. Figure generated with BioRender.com (License OT27PC68YH). (**F**–**H**) Representative immunoblots (**F**) and relative quantifications (**G** and **H**) revealed significantly increased total uncleaved junctophilin-2 (JPH2), but not cleaved JPH2 NT1, in *Obscn-ΔIg5859* atria compared with wild-type at 12 months; *t* test, *P < 0.05; *n* = 3 animals per group (3.5 and 6 months), *n* = 6 animals per group (12 months). Numbers on right of blots represent kilodaltons; data points represent the average of at least 3 technical replicates per animal.

We therefore investigated the levels, phosphorylation profile, and localization of T-cap in *Obscn-*Δ*Ig58/59* atria. At 6 months, T-cap expression was significantly increased in *Obscn-*Δ*Ig58/59* atria compared with age-matched controls, whereas T-cap levels were unaltered at 12 months (Figure 5, A and B). Due to the lack of commercial antibodies for pSer157 and pSer161, we utilized Phos-tag acrylamide gels to separate the different T-cap phospho-species. We detected a biphosphorylated (2P) species, 2 monophosphorylated forms (1P_1_ and 1P_2_), and nonphosphorylated T-cap (0P; Figure 5C). Although the 1P_1_ and 1P_2_ species harbor the same number of phosphates, they exhibit distinct mobilities, since Phos-tag electrophoresis may differentially delay the migration of proteins depending on not only the number but also the location of phosphate groups (33). Following normalization to total T-cap levels, we did not observe a significant difference in the abundance of any phospho-species between wild-type and *Obscn-*Δ*Ig58/59* atria at 6 months (Figure 5D). However, at 12 months, *Obscn-*Δ*Ig58/59* atria exhibited a significant upregulation of the 2P species accompanied by a corresponding decrease in the lower molecular weight 1P_1_ species compared with age-matched wild-type (Figure 5, D and E), a finding that is in agreement with the reduced phosphorylation levels of Ser161 detected in our phosphoproteomics screen (17). Despite its altered expression at 6 months or phosphorylation at 12 months, T-cap was properly localized to sarcomeric Z-disks in *Obscn-*Δ*Ig58/59* atria at both 6 and 12 months (Supplemental Figure 2).

Previous studies have postulated that T-cap expression is augmented as an adaptive response to sustained cardiac stress (31). To determine whether dysregulation of T-cap expression and phosphorylation is a direct consequence of the Ig58/59 deletion or secondary to maladaptive structural and electrical remodeling, we examined T-cap and pT-cap levels in the atria of 3.5-month-old *Obscn-*Δ*Ig58/59* mice, at a stage immediately prior to the onset of arrhythmia, remodeling, and dysfunction (16). There was no significant difference in total T-cap expression between genotypes at 3.5 months of age (Figure 5, A and B). However, quantification of T-cap phosphorylation via Phos-tag immunoblotting revealed a significant increase in 2P T-cap and a corresponding decrease in 1P_1_ T-cap in 3.5-month-old *Obscn-*Δ*Ig58/59* atria compared with age-matched controls, reminiscent of the T-cap phospho-spectra in 12-month *Obscn-*Δ*Ig58/59* atria (Figure 5, C and D). The 0P species was not reliably detected in lysates from 3.5-month-old atria and was therefore not included in quantifications. Thus, the altered phosphorylation profile of T-cap appears to be an early consequence of the Ig58/59 deletion, which becomes obscured at 6 months due to a compensatory upregulation of total T-cap and is reexposed and exacerbated at 12 months. Collectively, these findings indicate that deletion of Ig58/59 leads to intrinsic molecular changes in the phosphorylation profile of T-cap, possibly contributing to the maladaptive remodeling of the Z-disk and TAT membranes in *Obscn-*Δ*Ig58/59* atria.

### The expression of junctophilin 2 is altered in Obscn-ΔIg58/59 atria.

Given the substantial degradation of TAT structures in *Obscn-ΔIg58/59* atria, we next queried whether Ig58/59 deletion altered the SR network, too. Previous analysis of *Obscn-ΔIg58/59* atrial lysates revealed no changes in the expression of SR proteins SERCA, RyR2, or small ankyrin 1 (sAnk1) (17). Similarly, sAnk1 localization was not different in *Obscn-ΔIg58/59* atria compared to wild-type controls, suggesting that the SR structure is unaffected by Ig58/59 ablation (Supplemental Figure 3). However, we identified increased expression of the full-length form of junctophilin 2 (JPH2) in *Obscn-ΔIg58/59* atria at 12 months (Figure 5, F and G). JPH2 fastens the transverse tubules to the SR in cardiomyocytes and dictates the dimensions of the dyadic cleft (34). In fact, JPH2 downregulation is a common corollary of TAT remodeling in heart disease (35). Yet, calpain cleavage of JPH2 yields an approximately 75 kDa N-terminal fragment, JPH2 NT1, that is commonly upregulated under conditions of cardiac stress (36, 37), though not following Ig58/59 ablation (Figure 5H). JPH2 NT1 translocates to the nucleus, where it acts as a cardioprotective transcription factor, governing genes involved in hypertrophy, fibrosis, and inflammation (37). As there is a necessary trade-off between the TAT-tethered population of JPH2 critical for excitation-contraction coupling and the nuclear pool of cleaved JPH2 NT1, a relative increase in the noncleaved form of JPH2 in 12-month *Obscn-ΔIg58/59* atria may act as an adaptive measure to reinforce remaining dyads at the expense of inducing a protective gene program. In agreement, despite the loss of TATs in *Obscn-ΔIg58/59* atria, we detected no changes in JPH2 localization via immunofluorescence (Supplemental Figure 4). In sum, although the SR appears unchanged in *Obscn-ΔIg58/59* atria, by 12 months of age, there is notable dysregulation of the proteins involved in linking TATs to both the sarcomeric cytoskeleton (T-cap) and the SR (JPH2).

## Discussion

**Figure 6:**
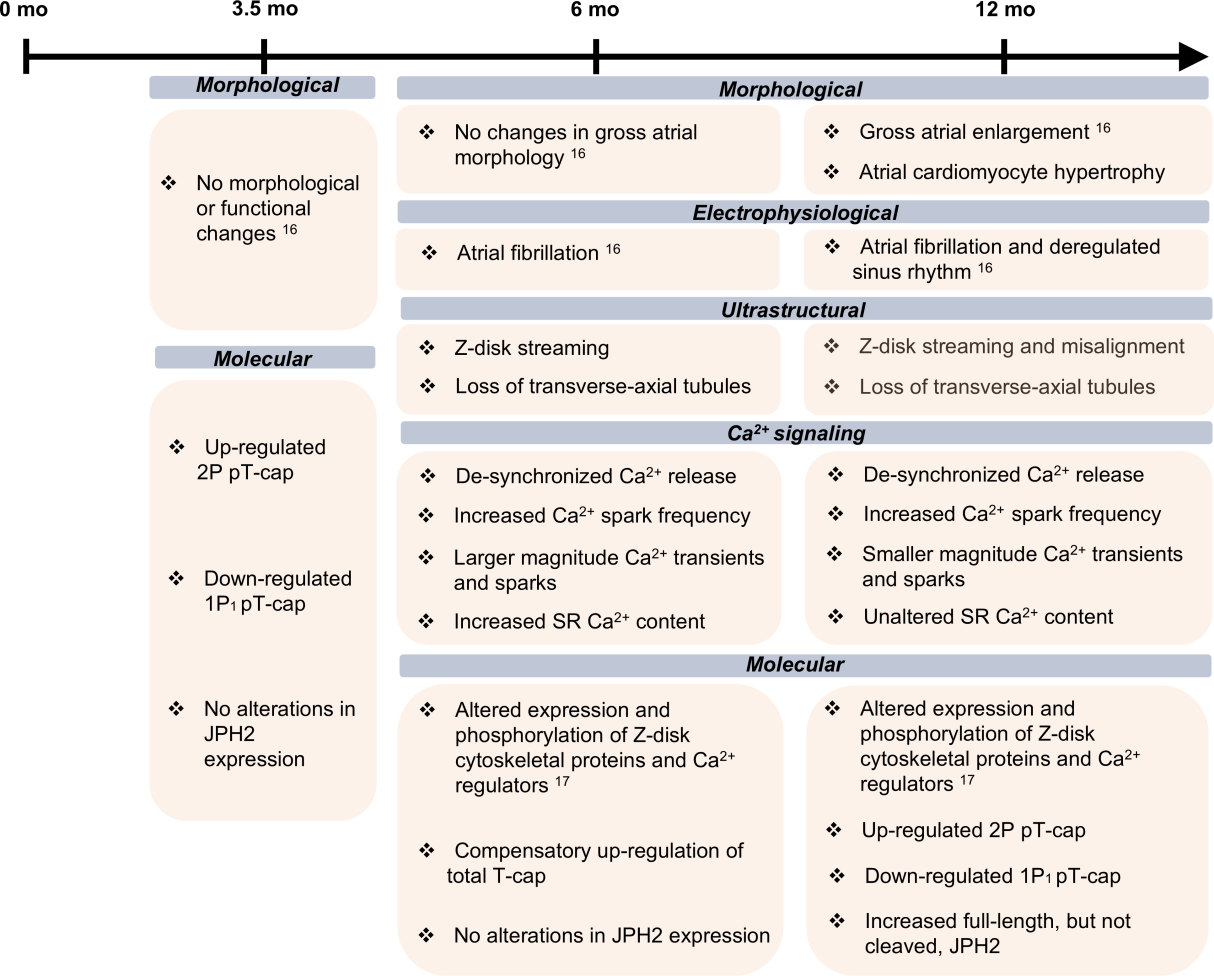
The *Obscn-ΔIg58/59* atrial phenotype. The phosphorylation profile of T-cap is dysregulated prior to the onset of arrhythmia and cardiac remodeling (16) in *Obscn-ΔIg58/59* atria at 3.5 months. Between the ages of 6 and 12 months, *Obscn-ΔIg58/59* mice undergo progressive structural remodeling and Ca^2+^ dysregulation accompanied by changes in T-cap expression and phosphorylation (17) as well as JPH2 expression that align with the onset and progression of AF.

Our current findings in combination with our prior proteomics study (17) provide insights into the cellular and molecular alterations underlying atrial remodeling and arrhythmia in *Obscn-*Δ*Ig58/59* mice (Figure 6). Specifically, our ultrastructural analysis indicated that deletion of the Ig58/59 module significantly affected the orientation and alignment of Z-disks in atria. In accordance with this, our proteomic analysis of *Obscn-*Δ*Ig58/59* atria exposed extensive alterations in the expression or phosphorylation status of Z-disk–associated cytoskeletal and regulatory proteins (17). The Z-disk is a critical nexus where the sarcomeric cytoskeleton interfaces with surrounding cellular structures, including internal membrane systems, the extra-sarcomeric cytoskeleton, intercalated discs, costameres/sarcolemma, and the TAT network (38, 39). Consequently, the Z-disk simultaneously governs a diverse array of cellular processes, such as sarcomeric assembly, force production, cell adhesion, intracellular Ca^2+^ homeostasis, and metabolism, while serving as a hotspot for mechanosensitive signaling pathways (38, 39). Thus, perturbations in Z-disk–associated protein complexes (17) likely render *Obscn-*Δ*Ig58/59* atria susceptible to routine mechanical stress, worsening Z-disk alignment and sarcomeric topography.

Accordingly, we identified age-specific alterations in the expression (6 months) and phosphorylation (3.5 and 12 months) status of the Z-disk–localized, titin-binding protein T-cap in *Obscn-*Δ*Ig58/59* atria. Prior to the onset of arrhythmia and whole-organ dysfunction (16), we observed reduced monophosphorylated (1P_1_) and increased biphosphorylated (2P) T-cap species, suggesting that altered T-cap phosphorylation develops as a direct consequence of Ig58/59 deletion, likely contributing to the initiation of Z-disk destabilization in *Obscn-*Δ*Ig58/59* atria. By 6 months, total T-cap expression is increased in *Obscn-*Δ*Ig58/59* atria, which may be an adaptation to insulate the Z-disk and associated structures from excessive mechanical strain. Such a notion would be in agreement with previous studies documenting that sustained exposure to cardiac stress prompts upregulation of T-cap expression (31). While 12-month *Obscn-*Δ*Ig58/59* atria do not exhibit this same compensatory increase in T-cap levels, we observed a reduction in a single monophosphorylated T-cap species, likely pSer161 (17), with a complementary increase in 2P T-cap, akin to what was found in sedentary 3.5-month-old *Obscn-*Δ*Ig58/59* atria. Thus, our findings indicate that deletion of obscurin-Ig58/59 induces alterations in the phosphorylation profile of the Z-disk–associated protein T-cap, which is concealed at 6 months due to adaptive upregulation of total T-cap and intensified at 12 months when Z-disk architecture and TAT network density appear dramatically deteriorated.

The obscurin-Ig58/59 module interacts with the NH_2_ terminal Ig9/10 domains of titin at the Z-disk within relative proximity to T-cap’s binding site on titin-Ig1/2 region (14, 29). While localization of T-cap to sarcomeric Z-disks appeared unaffected in *Obscn-*Δ*Ig58/59* atria, it is tempting to speculate that disruption of obscurin/titin binding via deletion of Ig58/59 may indirectly impact T-cap association with titin-Ig1/2. Alternatively, it may influence the ability of obscurin and titin to serve as molecular scaffolds for local kinase and phosphatase networks that regulate T-cap’s phosphorylation. Along these lines, our phosphoproteomics analysis revealed many Z-disk–localized and/or actin-associated cytoskeletal proteins with deregulated phosphorylation, including plectin, cortactin, synaptopodin 2-like, LIM-domain binding protein 3 (ZASP), myozenin, and synemin, in addition to T-cap (17). Intriguingly, our proteomics results also revealed an upregulation of Ca^2+^/calmodulin-dependent kinase II (CaMKII) phosphorylation at Thr331 in 12-month *Obscn-*Δ*Ig58/59* atria (17). Although the significance of this phosphorylation event is not yet understood, Thr331 resides within the CaMKII linker region along with a handful of other phospho-sites that putatively govern CaMKII autophosphorylation and activation (40). Given that T-cap Ser157 and Ser161 are substrates of CaMKII (28), it is possible that CaMKII Thr331 phosphorylation in 12-month *Obscn-*Δ*Ig58/59* atria could contribute to excess T-cap biphosphorylation in *Obscn-*Δ*Ig58/59* atria.

To date, the precise function of T-cap biphosphorylation remains undefined, though it has been suggested that constitutive phosphorylation of Ser157 and Ser161 regulates the overall stability of T-cap. Wirianto et al. previously reported that dually phospho-ablated exogenous T-cap is protected against proteasomal degradation when overexpressed in 293T cells (32). Contrary to this, Lewis et al. observed a robust decrease in ventricular, dually phospho-ablated, endogenous T-cap levels in the respective knockin mouse line (31). Given these discrepant findings along with the difficulty in disentangling the downstream effects of T-cap loss versus phospho-ablation, and the lack of knowledge regarding the potentially distinct functions of pSer157 and pSer161, our current understanding of the consequences of T-cap phosphorylation remains limited. Despite this lingering ambiguity in the literature, a clear link has been established among T-cap expression, T-cap phosphorylation, TAT structure, and the synchronicity of Ca^2+^ release (27, 28). Accordingly, ventricular myocytes isolated from T-cap–knockout mice exhibit a progressive loss of transverse tubule membranes, dyssynchronous Ca^2+^ release, and frequent Ca^2+^ sparks as they age (27). Moreover, overexpression of phospho-ablated T-cap, lacking both pSer157 and pSer161, in rat ventricular myocytes leads to disordered, but not diminished, transverse tubules along with prolonged, desynchronized Ca^2+^ release. Importantly, our current findings demonstrate that the putative roles of T-cap in regulating the integrity of the transverse tubule network and the synchronicity of Ca^2+^ release also apply to atrial cardiomyocytes.

The substantial depletion of the TAT system in *Obscn-*Δ*Ig58/59* atria may implicate a reduction in aligned RyR2/L-type Ca^2+^ channel (LTCC) junctional complexes that facilitate proper CICR. Just as orphaned (i.e., misaligned) RyR2 clusters are known to produce arrhythmogenic Ca^2+^ release in ventricular myocytes in heart failure (41), TAT depletion has been detected in atrial myocytes derived from sheep with AF (42). Relatedly, seminal work by Brandenburg et al. (43) demonstrated the importance of the TAT system and its orientation (axial versus transverse) for synchronous Ca^2+^ release in atrial myocytes. Specifically, these authors showed that atrial myocytes adapt to hypertrophy by increasing axial TAT elements thereby maintaining Ca^2+^ release despite maladaptive remodeling (43). No such compensation of the TAT system occurred in our model, despite upregulation of the junctional protein JPH2 at 12 months, which has been shown to restore TAT regression and enhance LTCC recruitment elsewhere (35). Consequently, it seems the structural deficits afflicting Z-disks and TATs in *Obscn-*Δ*Ig58/59* atria, driven by extensive alterations in the expression and phosphorylation of Z-disk–associated proteins including T-cap, are too systemic to be overcome by late-stage adaptive mechanisms (17). Instead, the progressive deterioration of the TAT network in *Obscn-*Δ*Ig58/59* atrial cardiomyocytes coincides with the development and advancement of arrhythmogenic Ca^2+^ handling dynamics. We thus posit that TAT depletion constitutes an important, emerging mechanism of Ca^2+^-based arrhythmogenicity and associated atrial cardiomyopathy.

*Obscn-*Δ*Ig58/59* male mice exhibit episodes of spontaneous arrhythmia reminiscent of human AF, with the frequency and severity of these episodes increasing as the mice age from 6 to 12 months (16). In line with this, we witnessed progressive abnormalities in Ca^2+^ cycling kinetics, Ca^2+^ sparks, and SR Ca^2+^ content. Specifically, at 6 months, we observed increased SR Ca^2+^ load associated with prolonged, amplified, and desynchronized Ca^2+^ transients that were accompanied by larger and more frequent Ca^2+^ sparks. While desynchronized Ca^2+^ release and elevated Ca^2+^ spark frequency persisted through 12 months, Ca^2+^ release amplitude and kinetics were substantially depressed. These changes are consistent with the natural progression of AF from paroxysmal to permanent (44). Indeed, previous work in atrial myocytes isolated from patients with paroxysmal AF revealed an increase in SR Ca^2+^ load akin to the phenotype of 6-month-old *Obscn-*Δ*Ig58/59* atria (45). Further, atrial myocytes isolated from a patient with chronic AF exhibited no alterations in SR Ca^2+^ load, similar to our findings in 12-month-old *Obscn-*Δ*Ig58/59* mice (46). Promiscuous Ca^2+^ spark activity may result from excessive RyR2 leak, frequently linked to hyperphosphorylation of RyR2 at Ser2808 and Ser2814. Our phosphoproteomic analysis of 12-month *Obscn-*Δ*Ig58/59* atria revealed increased phosphorylation of RyR2 at Ser2811, a CaMKII- and PKA-sensitive site within the “phosphorylation hotspot” not yet fully characterized but theorized to augment channel open probability (17, 47, 48). We also detected reduced phosphorylation of histidine rich Ca^2+^ binding protein — a regulator of SR Ca^2+^ uptake, storage, and release — at another functionally uncharacterized site, Ser272, in *Obscn-ΔIg58/59* atria at 12 months (17). Collectively, our data intimate a mechanism where deregulated Ca^2+^ dynamics in *Obscn-*Δ*Ig58/59* atria develop secondary to molecular alterations and structural deficits, which is corroborated by our proteomic screen in *Obscn-*Δ*Ig58/59* atria (17).

Ventricular and atrial tissues composing the different chambers of the heart possess inherent differences in cellular morphology, TAT membranes, and Ca^2+^ cycling (49, 50). Consistent with this, our current study provides evidence of distinct pathophysiological alterations in the atria versus ventricles (16) due to obscurin-Ig58/59 deletion. In particular, while the pathological manifestations of Ig58/59 ablation in ventricles are regulatory in nature (i.e., deregulated Ca^2+^ cycling due to changes in key Ca^2+^ cycling proteins in the absence of ultrastructural alterations) (16), *Obscn-ΔIg58/59* atria exhibit discrete and antecedent structural and signaling deficits (16). Critically, enhanced ventricular contractility in 6-month *Obscn-ΔIg58/59* male hearts, evidenced by increased ejection fraction and fractional shortening in the absence of ventricular myocyte abnormalities (16), implies elevated pressure on the atria during systole. This excess hemodynamic strain may contribute to the structural and functional remodeling of atrial cardiomyocytes in *Obscn-ΔIg58/59* males. However, our biochemical analysis revealed alterations in T-cap phosphorylation in *Obscn-*Δ*Ig58/59* atria as early as 3.5 months of age, prior to the development of ventricular remodeling at 6 months. We therefore postulate that obscurin and/or Ig58/59 may serve specialized roles in different cardiac chambers, rendering the atria particularly vulnerable to progressive pathophysiological remodeling due to Ig58/59 ablation.

Our current findings situate the *Obscn-*Δ*Ig58/59* mouse model as one of few surrogates for human ACM, featuring AF, atrial dilation, and progressive, sex-dependent pathogenesis. Not only do *Obscn-*Δ*Ig58/59* male atria mimic the morphological and electrophysiological consequences of this disease, but they also mirror the cellular and molecular hallmarks of ACM class 1, characterized by principal changes to the cardiomyocyte driven by genetic factors that culminate in lone AF in the absence of substantial fibrosis (1). In other genetic models featuring AF, the origin and accelerant of arrhythmogenesis is not always explicit. In the *Obscn-*Δ*Ig58/59* mouse model, we demonstrate a clear chronology wherein the onset and advancement of AF in males coincides with the progressive ultrastructural deficits and Ca^2+^ cycling dysfunction of atrial cardiomyocytes. Just as *Obscn-*Δ*Ig58/59* females are insulated against atrial remodeling and AF, women commonly incur AF later than men, often manifesting after menopause (51). The prominent sex differences in AF incidence in our model are likely driven by sex hormones, although estrogen, progesterone, and testosterone exert complex effects on ECG morphology and arrhythmia prevalence (51, 52). Importantly, not all animal models of AF recapitulate the sex bias seen in human AF, but the *Obscn-*Δ*Ig58/59* model could be utilized to elucidate sex-specific arrhythmogenic processes (53, 54).

Taken together, our past (17) and present studies reveal that deletion of obscurin-Ig58/59 in atria disrupts the expression and phosphorylation state of T-cap among other Z-disk–associated structural and signaling proteins. Using ultrastructural evaluation, high-resolution imaging of intracellular Ca^2+^ dynamics, and live-cell, super-resolution microscopy of TAT membranes, we show that deletion of the obscurin-Ig58/59 module underlies adverse structural remodeling of Z-disks and TAT membranes in atrial cardiomyocytes that likely fuels Ca^2+^ deregulation and arrhythmia. These findings provide mechanistic insights into the development of atrial remodeling and arrhythmogenesis and establish the *Obscn-*Δ*Ig58/59* line as a genetic model of ACM where atrial pathology develops prior to ventricular maladaptation.

## Methods

### Sex as biological variable.

The current study focuses on the functional and molecular deficits in *Obscn-*Δ*Ig58/59* male atria. We observed robust, progressive arrhythmias in *Obscn-*Δ*Ig58/59* males, with 50% and 83% developing arrhythmia at 6 and 12 months, respectively, of which 37.5% and 83% experience AF (16). In contrast, 33% of 6-month and 37% of 12-month-old *Obscn-*Δ*Ig58/59* females exhibited 1 or more forms of arrhythmia under sedentary conditions (16). Interestingly, AF incidence actually declined over time in *Obscn-*Δ*Ig58/59* females, as 33% versus 12% displayed AF at 6 and 12 months, respectively. Female sex hormones, particularly estrogen, most likely insulate *Obscn-*Δ*Ig58/59* females from atrial remodeling and electrical abnormalities. Consequently, the current study focuses on the molecular mechanisms of atrial pathogenesis in *Obscn-*Δ*Ig58/59* male atria.

### Obscn-ΔIg58/59 constitutive deletion mice.

The *Obscn-*Δ*Ig58/59* constitutive deletion model was generated as described previously (GenOway, Lyon, France) (16). Genotypes were confirmed by polymerase chain reaction utilizing 2 distinct primer sets (16). All experiments were performed with homozygous male *Obscn-*Δ*Ig58/59* animals and age-matched male wild-type. Backcrossing of the *Obscn-*Δ*Ig58/59* colony is performed every 5–10 generations to protect against genetic drift.

### Hydroxyproline assay.

Hydroxyproline content was quantified from flash-frozen cardiac tissue as described previously (13, 16). Briefly, right and left atria were combined and boiled overnight in 0.2 mL of 6 mol/L HCl at 110°C. The hydrolyzed tissue was diluted 1:16 in isopropanol, combined 2:1 with Reagent A (62 mmol/L chloramine-T, 0.56 mol/L sodium acetate, 0.14 mol/L citric acid, 0.35 mol/L NaOH, and 30.8% v/v isopropanol in water), and incubated at room temperature for 5 minutes. Samples were further diluted 1:4 in Reagent B (0.35 mol/L *p*-dimethylbenzeldehyde, 17.55% v/v ethanol, and 1.19% v/v sulfuric acid in isopropanol), incubated at 55°C for 1 hour, and quenched on ice. Absorbance values were obtained at 558 nm, and hydroxyproline content was calculated using a standard curve and presented as either the absolute hydroxyproline concentration (μM) or normalized to input atrial tissue mass (μM/mg).

### Electron microscopy.

Atrial samples were prepared for electron microscopy following methods for mega metal staining (55). Briefly, atria isolated from 6- and 12-month-old wild-type and *Obscn-*Δ*Ig58/59* mice were fixed in 2% paraformaldehyde, 2.5% glutaraldehyde, and 0.1 mol/L PIPES buffer (pH 7.4); washed with 0.1 mol/L PIPES buffer; and postfixed for 60 minutes in 0.75% potassium ferrocyanide and 1% osmium tetroxide in 0.1 M PIPES buffer, followed by washing with water and 20 minutes of treatment with 1% freshly prepared thiocarbohydrazide solution at room temperature. After extensive washing with water, samples were stained with 1% osmium tetroxide for 60 minutes, washed in water, and left in 1% uranyl acetate overnight at 4^o^C. Samples were then stained with lead aspartate at 60°C for 30 minutes, washed with water, and dehydrated using serial graded ethyl alcohol (30%, 50%, 70%, 80%, 90%, and 100%) and 100% acetone. Samples were then embedded in Durcupan resin following the manufacturer’s recommendation (Electron Microscopy Sciences). Ultrathin sections at 70 nm thickness were cut on a UC6 ultramicrotome (Leica Microsystems, Inc.), then examined under a Tecnai T12 transmission electron microscope (Thermo Fisher Scientific) operated at 80 kV. Images were acquired with a bottom-mount charge-coupled device camera and AMT600 software (Advanced Microscopy Techniques). All samples were prepared and imaged at the Electron Microscopy Core Imaging Facility of the University of Maryland Baltimore.

Z-disk streaming was evaluated by annotating the presence or absence of streaming in a subset of images taken at 3,200× original magnification (10 ± 3 images per animal at 6 months, 15 ± 3 images per animal at 12 months). A 2-sided Fisher’s exact test (GraphPad Prism software 5.00) was used to compare the proportion of images containing Z-disk streaming between age-matched wild-type and *Obscn-*Δ*ig58/59* mice. Variability in Z-disk orientation was evaluated by calculating the absolute deviation of the Z-line angle (measured with ImageJ, NIH) for all sarcomeres visible in 2 representative images per animal (taken at 3,200× original magnification; approximately 20–80 sarcomeres were analyzed per image).

### Atrial cardiomyocyte isolation.

Atrial cardiomyocytes were isolated from 6- and 12-month-old mice using a modified Langendorff perfusion system as described previously (16, 24, 56, 57). Mice were anesthetized using 3% isoflurane in oxygen and injected intraperitoneally with 108 U heparin. Dissected hearts were placed directly in digestion buffer (DB; 133 mmol/L NaCl, 5 mmol/L KCl, 2 mmol/L MgCl_2_•6H_2_O, 1.2 mmol/L KH_2_PO_4_, 6 mmol/L taurine, 6 mmol/L creatine, 10 mmol/L glucose, 10 mmol/L HEPES, pH 7.4) containing 0.4 mmol/L EGTA (DB-EGTA) on ice. Hearts were cannulated through the aorta and perfused in retrograde with DB-EGTA for 2 minutes and subsequently perfused with DB-Enzymes solution containing 4 mg/mL bovine serum albumin (BSA), 0.3 mmol/L CaCl_2_, 1 mg/mL collagenase (Worthington), 0.04 mg/mL trypsin (MilliporeSigma), and 0.04 mg/mL protease type XIV (MilliporeSigma P5147) for 5 minutes at 37°C. Atria were separated from ventricles, minced, and subjected to additional digestion in DB-Enzymes for 5 minutes at 37°C. Enzymatic digestion was terminated by transferring atrial tissues to DB containing 4 mg/mL BSA, 3.2 mg/mL 2,3-butanedione monoxime, and 0.2 mmol/L CaCl_2_, where myocytes were mechanically dispersed by trituration with a Pasteur pipette. Only myocytes that exhibited appropriate morphology (rod-shaped with clear cross striations) and were responsive to electrical stimulation were used for downstream experimentation. Given the technical difficulties involved in isolating high-quality, primary atrial cardiomyocytes from a miniscule amount of tissue (<10 mg), any atrium yielding at least 2 healthy myocytes was included in analyses. Cells isolated from the same atria are similarly colored within figures, and the number of cells analyzed per atrial sample is indicated in the corresponding figure legends.

### Ca^2+^ imaging and analysis.

Ca^2+^ imaging in atrial myocytes was performed as described previously (24). Isolated cardiomyocytes were plated in chambers coated with ECM gel (e1270, MilliporeSigma) and mounted on a Nikon Eclipse Ti inverted microscope with a 60× Oil 1.4 NA objective. Cells were loaded for 20 minutes with 1 μmol/L fluo-4-acetoxymethyl ester (Fluo-4-AM; Thermo Fisher Scientific F14201) followed by de-esterification for 10 minutes. Subsequently, cells were brought to physiological Ca^2+^ by perfusion with normal Tyrode’s solution (135 mmol/L NaCl, 5.4 mmol/L KCl, 1 mmol/L MgCl_2_•6H_2_O, 0.33 mmol/L NaH_2_PO_4_, 11 mmol/L glucose, 5 mmol/L HEPES, 1.8 mmol/L CaCl_2_, pH 7.4). Ca^2+^ transients were measured during 1 Hz external field stimulation (2 ms, 20 V; MyoPacer, IonOptix) using the 488 nm laser line of a confocal laser scanning microscope (Nikon A1R). Line scans (1.872 ms/line) were collected in transverse orientation for 30 seconds. Ca^2+^ sparks were imaged for 30 seconds in quiescent atrial myocytes preceded by 30 seconds of external field stimulation at 1 Hz to ensure steady-state SR Ca^2+^ loading. SR Ca^2+^ content was measured by rapid application of 10 mmol/L caffeine to quiescent cells preceded by steady-state external field stimulation at 1 Hz for 30 seconds. Field stimulation was subsequently restarted at 1 Hz to ensure that all releasable Ca^2+^ had been depleted from the SR.

The resulting electrically or caffeine-induced Ca^2+^ transients were analyzed offline using ImageJ and Clampfit analysis software v11.1 (Molecular Devices). Ca^2+^ transients were analyzed by averaging the Fluo-4 signal across the entire cell’s width and are presented as background-subtracted, normalized fluorescence (F/F_0_, arbitrary units). The delay of Ca^2+^ release across the transverse axis of the cardiomyocyte was evaluated using a custom-made Python script (58) quantifying the TTF_50_ for each pixel. The dispersion of delay values (i.e., SD) and the coefficient of variation (SD/mean) for each transient represent Ca^2+^ release synchrony. Ca^2+^ spark frequency and morphology were assessed from concatenated line scan images using ImageJ Sparkmaster plugin (59). Spark mass was calculated as amplitude × 1.206 × FWHM^3^ as described previously (60). 3-Dimensional surface plot renderings of Ca^2+^ sparks were generated with ImageJ.

### TAT imaging and analysis.

TAT imaging was carried out with a Zeiss LSM 880 confocal microscope equipped with an Airyscan super-resolution imaging module using a 63/1.40 Plan-Apochromat Oil differential interference contrast M27 objective lens (Zeiss) as described previously (61). Freshly isolated atrial cardiomyocytes were loaded with the membrane dye di-8-ANEPPS (5 μmol/L) and imaged within 1.5 hours after cell isolation as described previously (24). Only structurally intact atrial cardiomyocytes with continuous cell membranes were selected for analysis. For TAT analysis, the NIH open-source FIJI platform was used (62). Cell interior regions of interest (ROIs) were drawn with the polygon selection tool such that di-8-ANEPPS signal on the cardiomyocyte surface was excluded. These ROIs were processed using a FIJI macro derived from a previous report (63) and optimized for atrial TAT networks in older animals (Supplemental Data 1).

Branch lengths, branch counts, junction counts, directionality histograms, ROI areas, and micron-to-pixel ratios were saved in Excel files for each cell. These files were batch-analyzed using a custom Matlab script (25, 64). The proportion of transverse and axial tubules in each cell was calculated from the area under the curve of the directionality histograms at 90° ± 2° (transverse orientation) and 0° ± 2° (axial orientation).

### Lysate preparation, standard and Phos-tag gel electrophoresis, and immunoblotting.

Atrial lysates were generated as described previously (16, 17) from mice at 3–4 (denoted as 3.5 in the text), 6, or 12 months of age. Briefly, flash-frozen right and left atrial tissues were combined and ground into a fine powder using a glass Dounce homogenizer submerged in liquid nitrogen. The ground tissues were incubated at –20°C for 20 minutes and then solubilized in a 1:1 mixture of urea-thiourea lysis buffer (8 mol/L urea, 2 mol/L thiourea, 3% SDS, 0.05 mol/L Tris-HCl, 0.03% bromophenol blue, 0.075 mol/L dithiothreitol, pH 6.8) and 50% glycerol supplemented with protease and phosphatase inhibitors (Halt Protease and Phosphatase Inhibitor Cocktail, Thermo Fisher Scientific) in a 60°C water bath. Following centrifugation at 15,000*g*, the supernatant was collected, aliquoted, and flash-frozen in liquid nitrogen. Lysates were thawed at 55°C for 5 minutes and separated either by standard SDS-PAGE as described previously (16, 17) or by Phos-tag gel electrophoresis. For Phos-tag gel electrophoresis lysates were separated for 3 hours (30 mA/gel) on 12% polyacrylamide gels supplemented with 50 μmol/L Phos-tag acrylamide according to the Zn^2+^ Phos-tag (FUJIFILM Wako Chemicals) SDS-PAGE protocol according to the manufacturer’s instructions. Standard SDS-PAGE and Phos-tag gels were transferred to nitrocellulose membranes, blocked in 3% BSA, and probed with primary antibodies against T-cap (1:1,000; ab133646; Abcam), JPH2 (1:1,000; INV-405300; Invitrogen), GAPDH (1:15,000; G8795; MilliporeSigma), or α-actinin (1:1,000; A7811; MilliporeSigma). HRP-conjugated secondary antibodies (goat anti-mouse IgG, CST-7076S, or goat anti-rabbit IgG, CST-7074S; 1:3,000; Cell Signaling Technology) and chemiluminescence reagents (Pierce, ECL) were applied to visualize immunoreactive bands. Total T-cap was quantified via densitometry (ImageJ) and normalized to GAPDH or α-actinin as a loading control. The relative abundance of each T-cap phospho-species was determined by dividing the relative intensity of each species by the summed intensity of all species for a given sample and is presented as the percentage of total T-cap.

### Immunostaining and confocal microscopy.

Frozen cardiac sections were prepared as described previously (16). In brief, following perfusion and fixation in 2% paraformaldehyde in phosphate-buffered saline (PBS), dissected atria were embedded in 7.5% gelatin and 15% sucrose in PBS and frozen with 2-methylbutane. Samples sectioned at a thickness of 12 μm were permeabilized with 0.1% (T-cap) or 0.3% (JPH2 and sAnk1) Triton X-100 in PBS, blocked in 1 mg/mL BSA with 1 mmol/L sodium azide in PBS (T-cap) and 1% goat serum (JPH2 and sAnk1), and incubated with primary antibodies targeting T-cap (1:250; ab133646; Abcam), JPH2 (1:100; INV-405300; Invitrogen), or sAnk1 (1:200; ARP42566_T100; Aviva). Samples were then incubated with Alexa Fluor 488 goat anti-rabbit (1:300; A11034, Invitrogen) secondary antibody for 2 hours, stained with Alexa Fluor 647 phalloidin (1:30; A22287, Invitrogen) for 30 minutes, and mounted with VECTASHIELD mounting medium (Vector Laboratories). Immunostained sections were analyzed under a Nikon Spinning Disc confocal microscope at the University of Maryland School of Medicine (UMSOM) Confocal Microscopy Facility. The brightness/contrast of images was adjusted uniformly across the entire image.

### Statistics.

Statistical significance between age-matched wild-type and homozygous male *Obscn-ΔIg58/59* groups was determined by 2-tailed Student’s *t* test in all experiments, excluding those depicted in Figure 1D. A Fisher’s exact test was used in Figure 1D to compare Z-disk streaming. Error bars represent average values ± SEM. Sample sizes, cell and animal numbers, along with the statistical tests and *P* values for each experiment are noted in the corresponding figure legends. GraphPad Prism was used to calculate statistical parameters; *P* < 0.05 was considered statistically significant.

### Study approval.

Animal care and procedures were conducted under protocols approved by the Institutional Animal Care and Use Committee at the UMSOM and in accordance with the NIH guidelines (*Guide for the Care and Use of Laboratory Animals*, National Academies Press, 2011).

### Data availability.

Original images and blots are provided in the corresponding supplemental file, and raw data values are supplied in the Supporting Data Values file.

## Author contributions

Conceptualization was done by AG, AB, and AKK; data curation was done by AG, AB, and MG; formal analysis was done by AG, AB, HCJ, LB, ADK, and MG; funding acquisition was done by AKK; investigation was done by AG, AB, HCJ, LB, ADK, and MG; methodology was developed by AG, AB, HCJ, LB, ADK, and MG; project administration was done by CWW, MG, and AKK; resources were provided by CWW, MG, and AKK; software was developed by HCJ, LB, and ADK; supervision was done by HCJ, MG, and AKK; validation was done by AG, AB, HCJ, LB, ADK, and MG; visualization was done by AG, AB, HCJ, and MG; writing of the original draft was done by AG and AB; and writing, review, and editing were done by AG, AB, HCJ, LB, ADK, CWW, MG, and AKK.

## Supplementary Material

Supplemental data

Unedited blot and gel images

Supporting data values

## Version 1 01/13/2025

In-Press Preview

## Version 2 02/24/2025

Electronic publication
