# Supplementary material for: Constitutive deletion of the obscurin-Ig58/59 domains induces atrial remodeling and Ca2+-based arrhythmogenesis
Source: JCI Insight. 2025 Jan 7;10(4):e184202. doi: 10.1172/jci.insight.184202 (PMC11949006; doi:10.1172/jci.insight.184202)
Supplement: Supplemental data [file jciinsight-10-184202-s030.pdf]

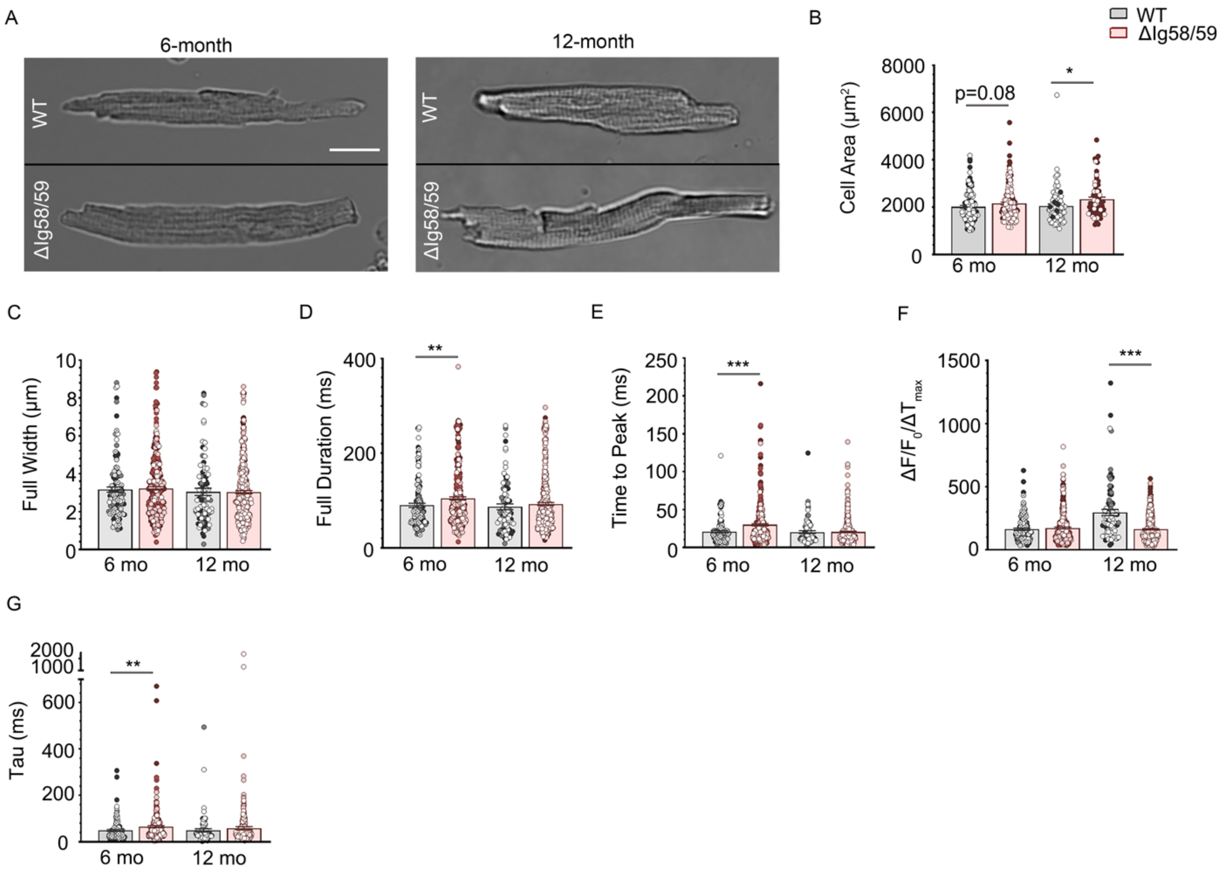

SFigure 1. Evaluation of Ca^{2+} spark morphology and Ca^{2+} in *Obscn-ΔIg5859* atria at 6- and 12- months. (A-B) Cardiomyocytes isolated from *Obscn-ΔIg58/59* atria were significantly enlarged at 12-months of age compared to age-matched wild-type; scale bar: 20 µm; t-test, *p<0.05; n=2 animals per group, 60-101 cells per heart (6-months), 7-83 cells per heart (12-months); data points represent individual cells and are color-coded by biological replicate. **(C-G)** Analysis of Ca^{2+} spark morphology revealed no significant differences in full width (C), and age-specific alterations in full duration (D), time to peak (E), the maximum steepness of spark upstroke calculated as $\Delta F/F_0/\Delta T_{max}$ (F), and the exponential time constant of decay, Tau (G) in *Obscn-ΔIg5859* atria at 6- and 12- months; t-test, **p<0.01***p<0.001, n= 5 animals per group (6-months), n=3 animals per group (12-months); 9-20 cells per heart (6-months), 7-17 cells per heart (12-months); data points represent individual sparks and are color-coded by biological replicate.

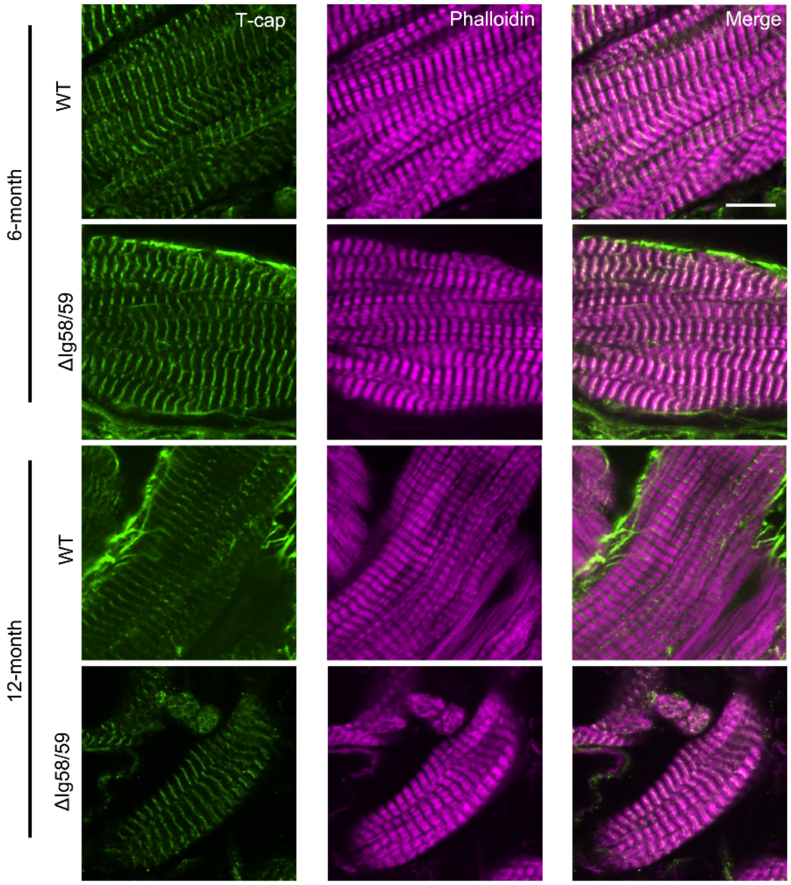

SFigure 2. The localization of T-cap is unchanged in *Obscn-ΔIg58/59* atria. Immunostained cryosections of wild-type and *Obscn-ΔIg5859* atrial tissues indicated that T-cap is properly localized to the Z-disk at both 6- and 12-months as determined by co-staining with the actin marker, phalloidin; scale bar: 10 µm.

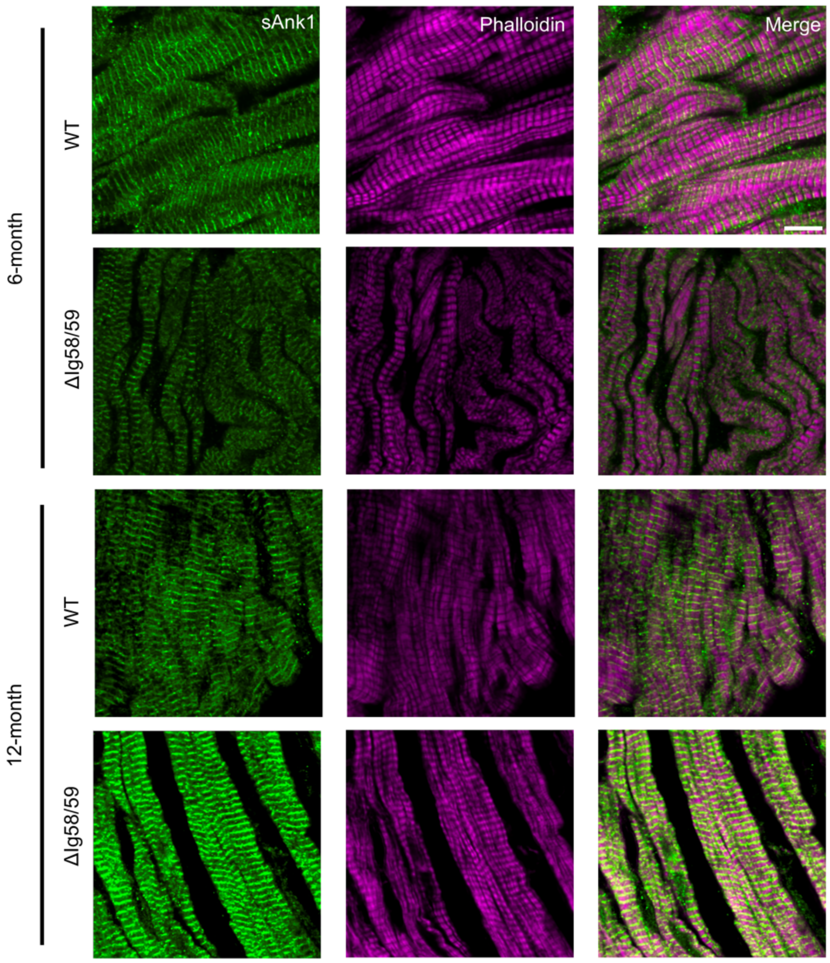

SFigure 3. The localization of sAnk1 is unchanged in *Obscn-ΔIg58/59* atria. Immunostained cryosections of wild-type and *Obscn-ΔIg5859* atrial tissues do not indicate alterations in sAnk1 localization at 6- or 12-months as determined by co-staining with the actin marker, phalloidin, suggesting the structure of the SR is unaffected in *Obscn-ΔIg5859* atria; scale bar: 10 µm.

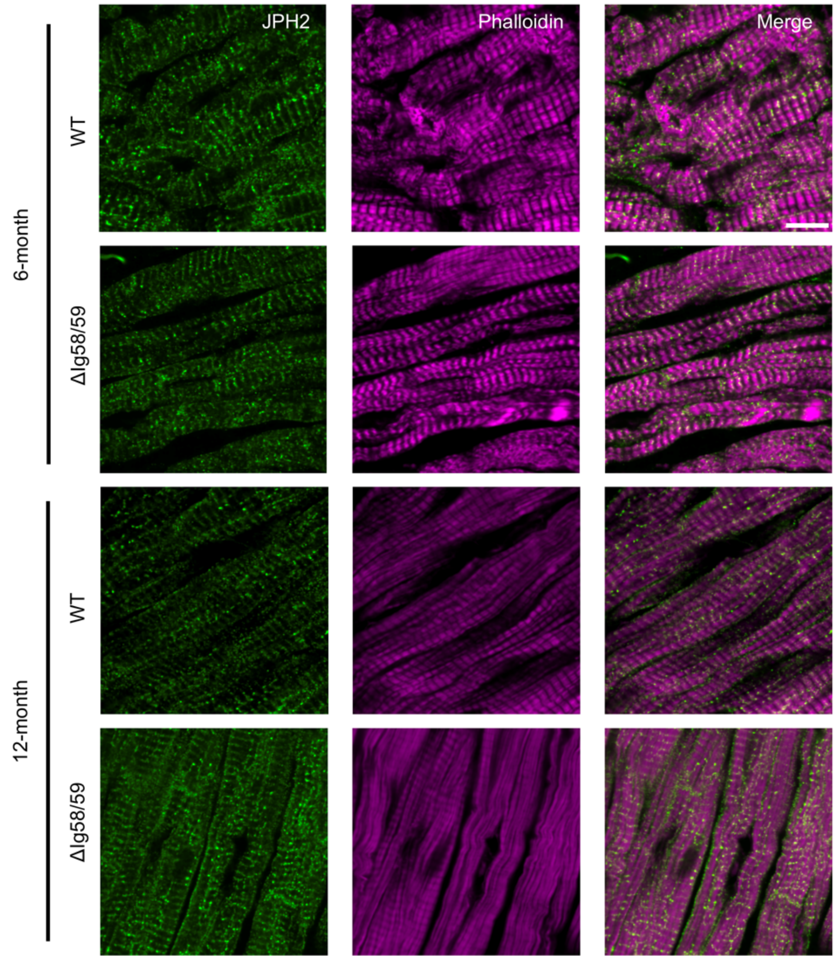

SFigure 4. The localization of JPH2 is unchanged in *Obscn-ΔIg58/59* atria. Immunostained cryosections of wild-type and *Obscn-ΔIg5859* atrial tissues do not indicate alterations in JPH2 localization at 6- or 12-months as determined by co-staining with the actin marker, phalloidin, suggesting that the junctional SR is unaffected in *Obscn-ΔIg5859* atria; scale bar: 10 µm.

Supplemental Data 1.

ROIs were processed using a FIJI macro derived from a previous report (63) and optimized for atrial TAT networks in older animals:

```
run(“Add to Manager”);
run(“Enhance Contrast”, “saturated=0.35”);
run(“Measure”);
run(“Duplicate…”, “ “);
run(“Clear Outside”);
run(“Subtract Background…”, “rolling=5”);
run(“8-bit”);
run(“Statistical Region Merging”, “q=100 showaverages”);
setThreshold(20, 255);
run(“Convert to Mask”);
run(“Skeletonize (2D/3D)”);
run(“Directionality”, “method=[Fourier components] nbins=180 histogram=-45 display_table”);
run(“Analyze Skeleton (2D/3D)”);
```
