# Supplementary material for: Constitutive deletion of the obscurin-Ig58/59 domains induces atrial remodeling and Ca2+-based arrhythmogenesis
Source: JCI Insight. 2025 Jan 7;10(4):e184202. doi: 10.1172/jci.insight.184202 (PMC11949006; doi:10.1172/jci.insight.184202)

Figure 1C

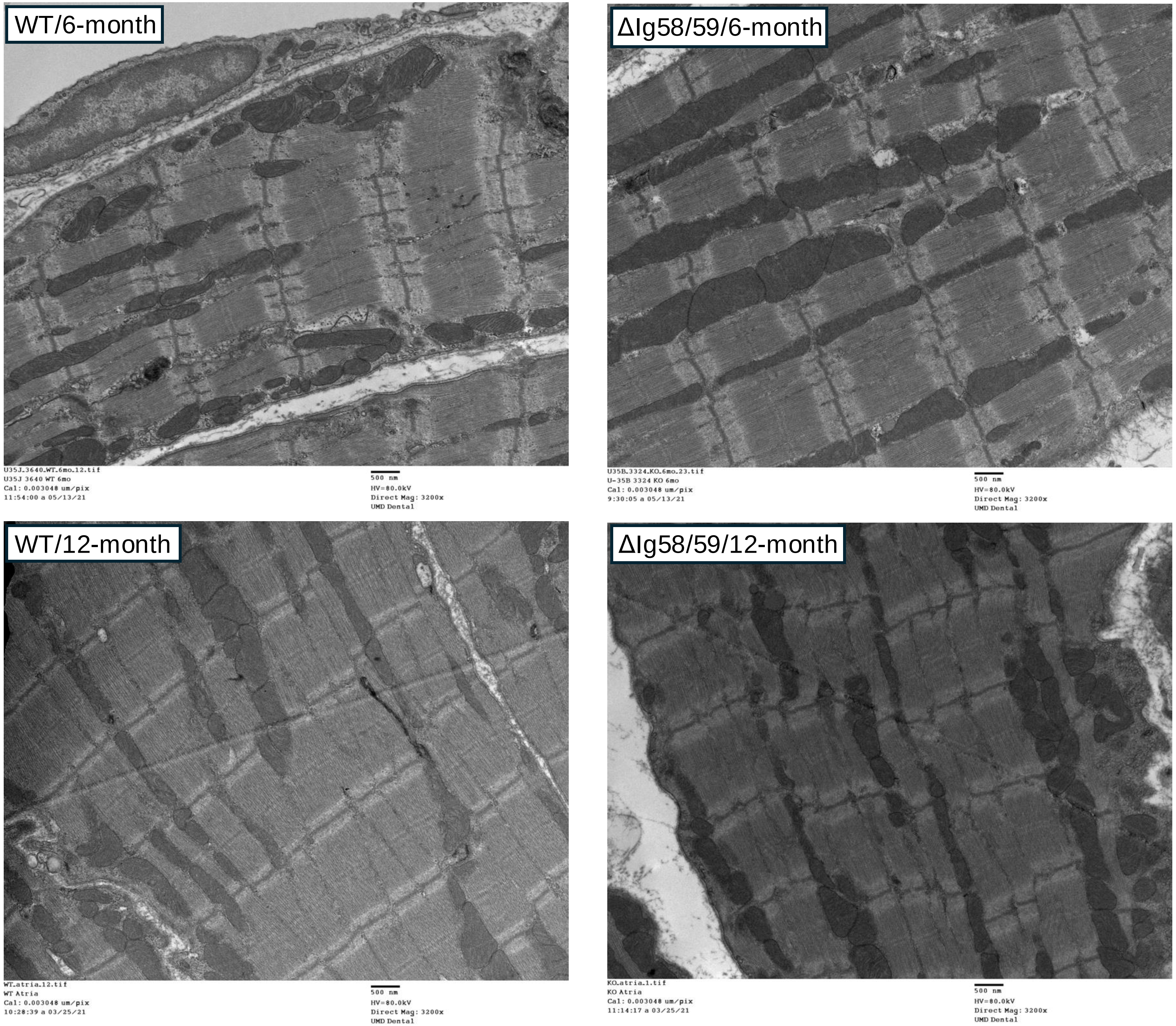
WT/6-month
U35J_3640_WT_6mo_12.tif
U35J 3640 WT 6mo
Cal: 0.003048 um/pix
11:54:00 a 05/13/21
500 nm
HV=80.0kV
Direct Mag: 3200x
UMD Dental
ΔIg58/59/6-month
U35B_3324_KO_6mo_23.tif
U-35B 3324 KO 6mo
Cal: 0.003048 um/pix
9:30:05 a 05/13/21
500 nm
HV=80.0kV
Direct Mag: 3200x
UMD Dental
WT/12-month
WT_atria_12.tif
WT Atria
Cal: 0.003048 um/pix
10:28:39 a 03/25/21
500 nm
HV=80.0kV
Direct Mag: 3200x
UMD Dental
ΔIg58/59/12-month
KO_atria_1.tif
KO Atria
Cal: 0.003048 um/pix
11:14:17 a 03/25/21
500 nm
HV=80.0kV
Direct Mag: 3200x
UMD Dental

Figure 4 A-B (Areas included in the figures are marked with a white box)

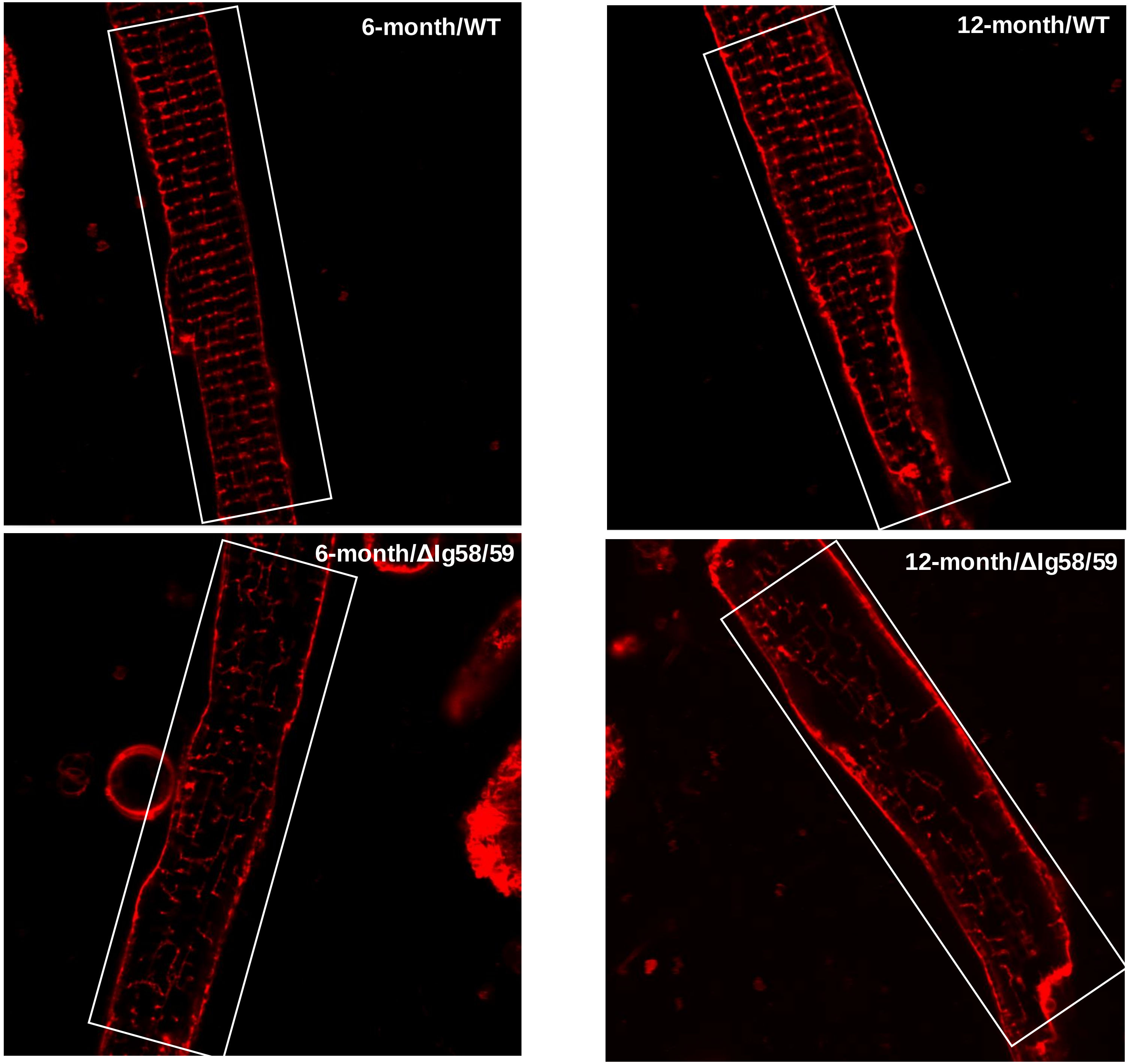

Figure 5 A/C (Blots are presented in grayscale mode in the figures; representative lanes used in the figures are marked with a red box)

Panel A

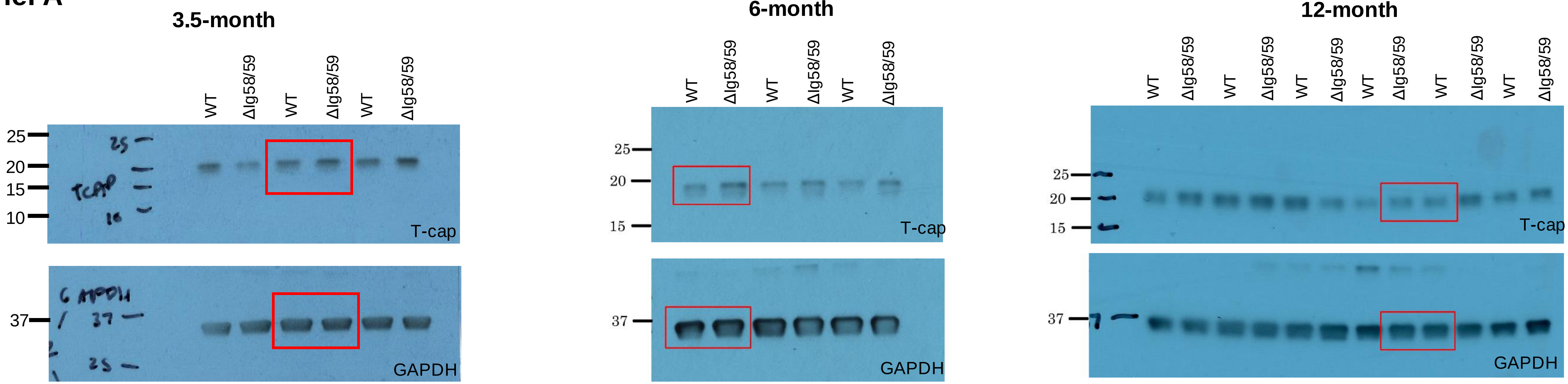

Panel C

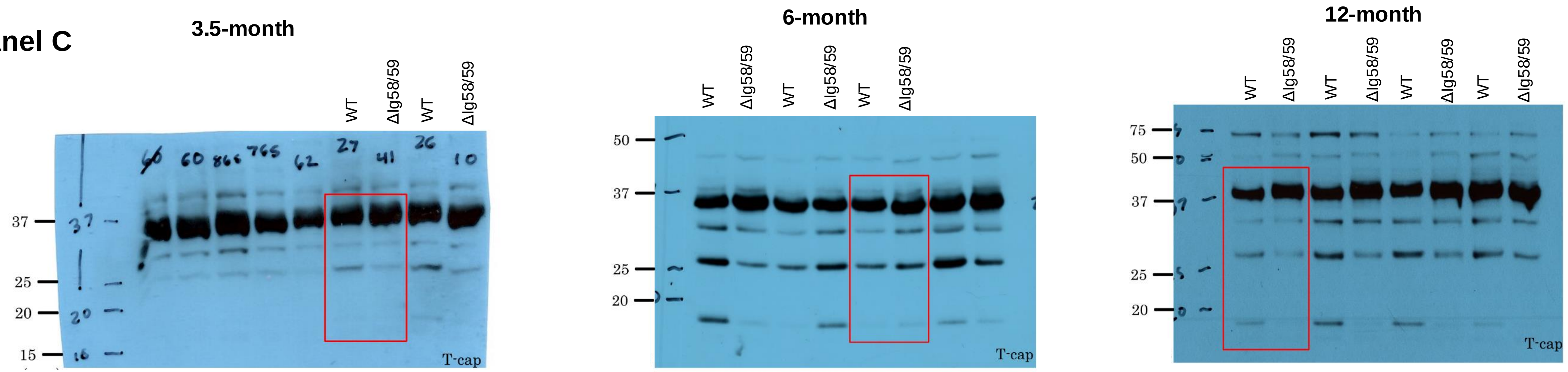

Figure 5F (Blots are presented in grayscale mode in the figures; representative lanes used in the figures are marked with a red box)

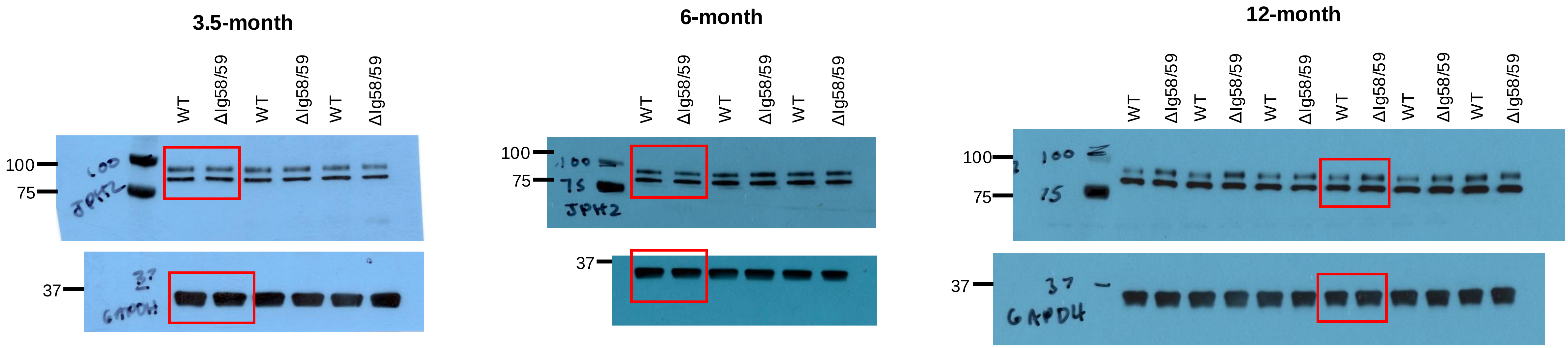

Supplemental Figure 2 (Areas included in the figures are marked with a white box)

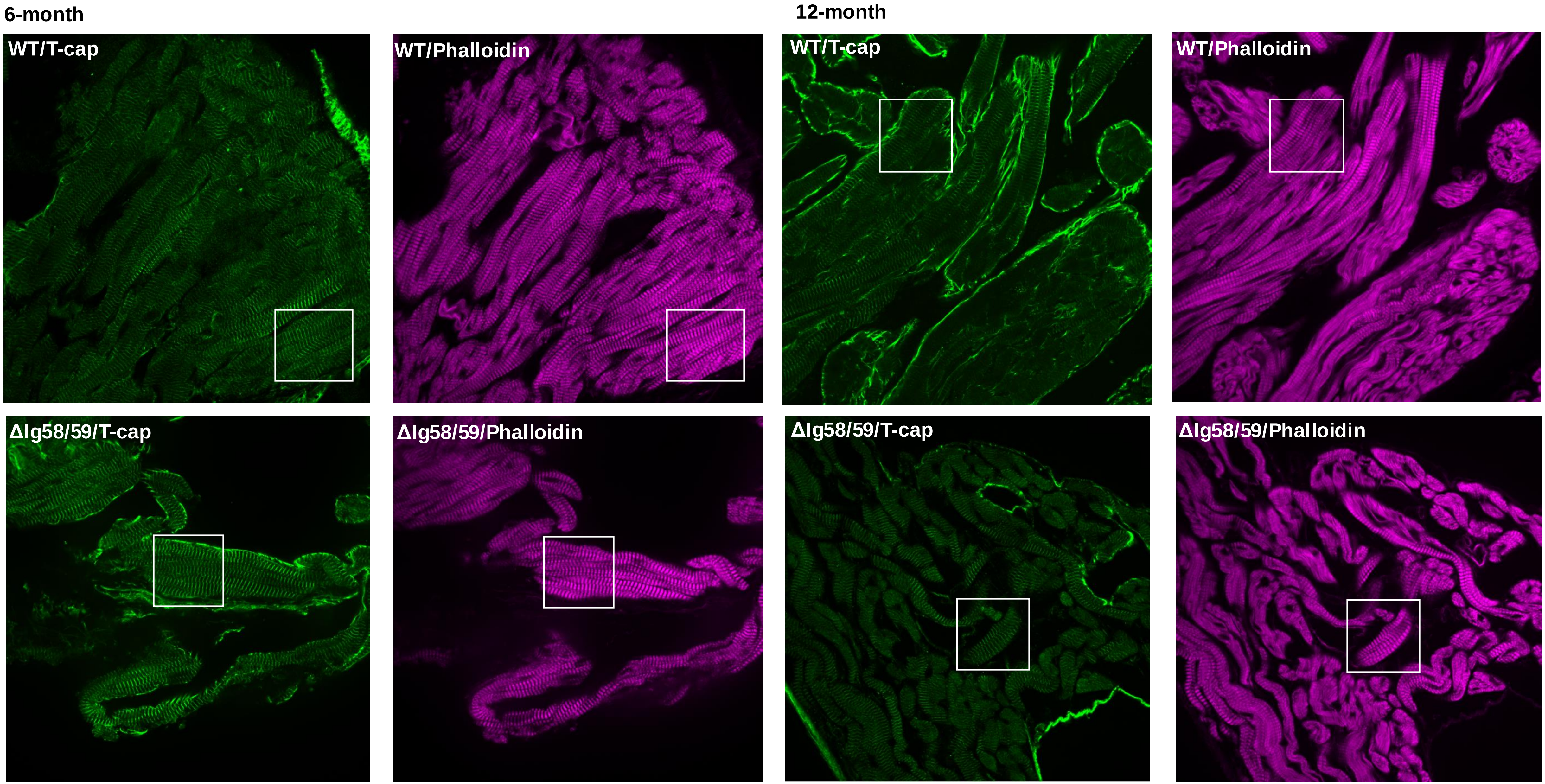

Supplemental Figure 3 (Areas included in the figures are marked with a white box)

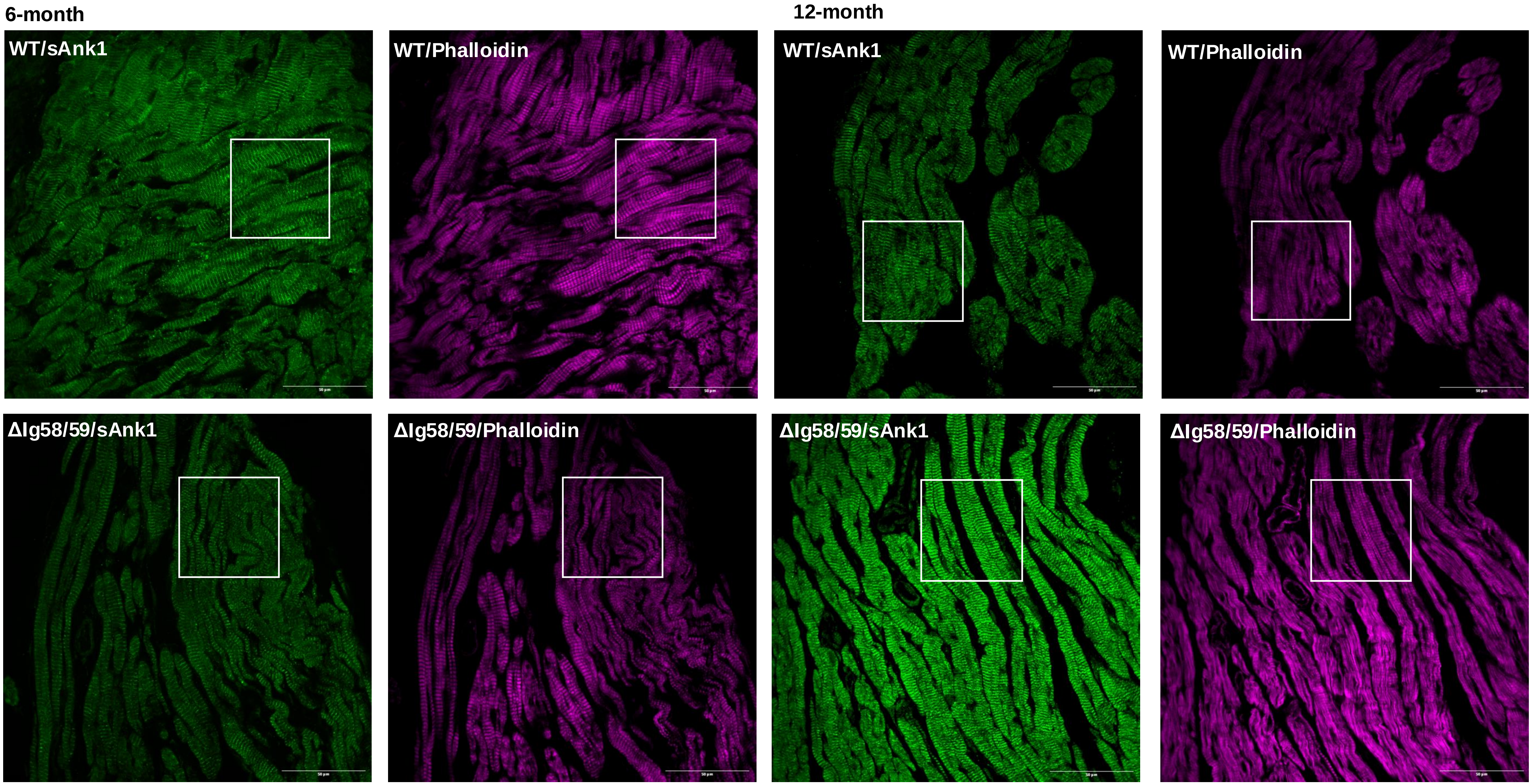

Supplemental Figure 4 (Areas included in the figures are marked with a white box)

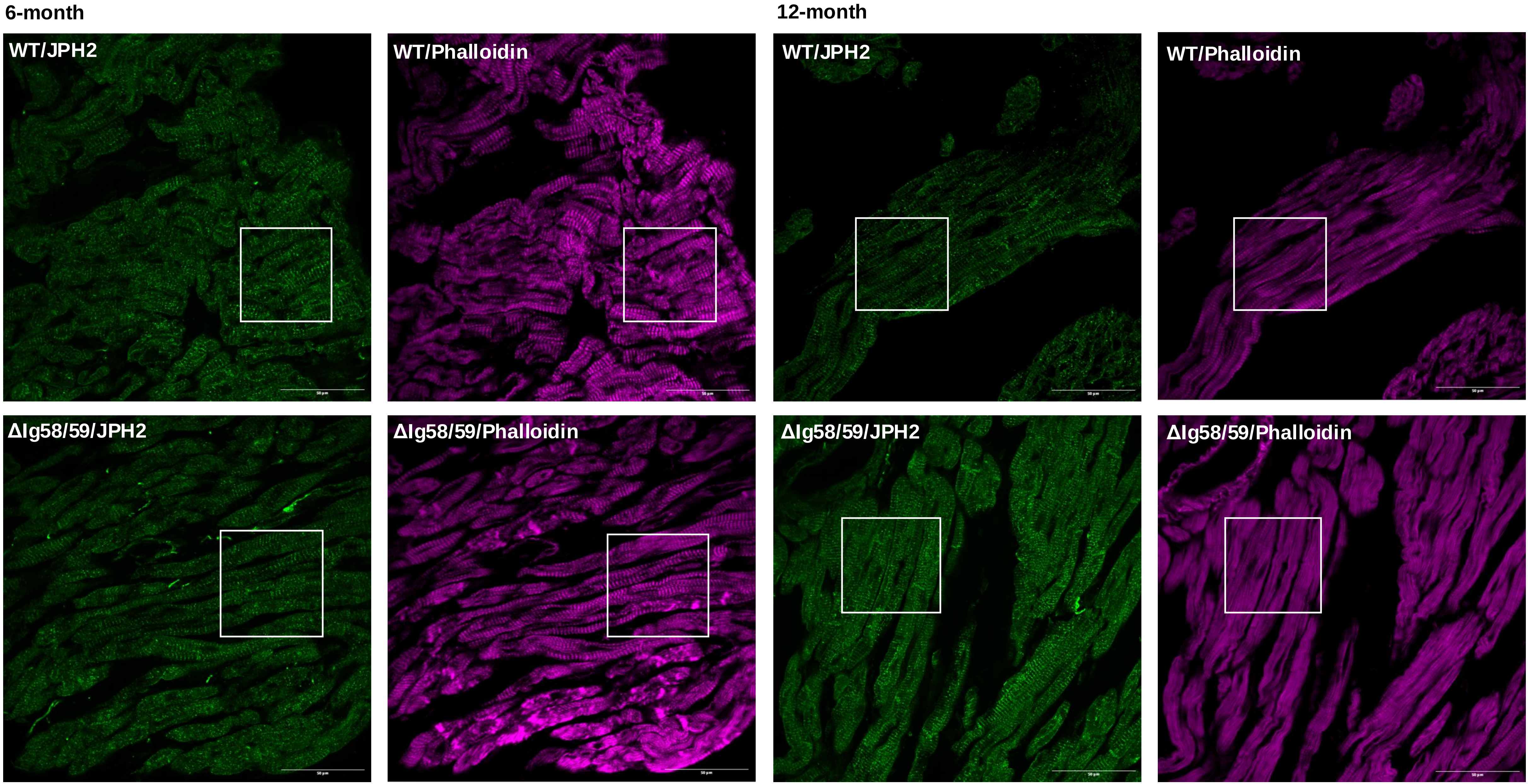

Supplement: Unedited blot and gel images [file jciinsight-10-184202-s031.pdf]
